# The Polymorphs of Diacetylcurcumin (DAC)

**DOI:** 10.3390/molecules31122133

**Published:** 2026-06-17

**Authors:** Marco A. Obregón-Mendoza, Rosario Tavera-Hernández, Rubén Sánchez-Obregón, Carolina Escobedo-Martínez, Rubén A. Toscano, Raúl G. Enríquez

**Affiliations:** 1 Instituto de Química, Universidad Nacional Autónoma de México, Mexico City 04510, Mexico; marco.obregon@zaragoza.unam.mx (M.A.O.-M.); rosario.tavera@gmail.com (R.T.-H.); rubens@unam.mx (R.S.-O.); toscano@unam.mx (R.A.T.); 2 Departamento de Farmacia, División de Ciencias Naturales y Exactas, Universidad de Guanajuato, Campus Guanajuato, Guanajuato 36050, Mexico; c.escobedo@ugto.mx

**Keywords:** polymorphism, diacetylcurcumin, DAC

## Abstract

Two new monoclinic and triclinic polymorphs of diacetylcurcumin are reported in addition to the previously known polymorph (monoclinic, space group *P*2_1_). These polymorphs result from different solvent mixtures that were successfully established. The solid-state NMR (CP-MAS) and X-ray studies allowed unambiguous authentication of polymorph 2 (canoe-shaped, *P*2_1_/*n*) and the concomitant polymorph 3 (balloon-shaped, *P*−1). We demonstrate that morphological crystal analysis under a microscope, combined with ATR-IR, is a rapid and inexpensive method for exploring the polymorphic landscape of curcuminoids. This contribution highlights the progress in curcumin derivative research and should inspire fellow chemists and materials scientists to explore further.

## 1. Introduction

Polymorphism has been widely recognised in the pharmaceutical industry [1] since the crystalline form of a material can indicate crucial physicochemical properties (such as melting point, solubility, and thermal stability) that are important for legal and patenting purposes. However, both basic and advanced research benefit from exploring polymorphism. Theoretically, understanding intermolecular interactions and crystal lattice energies [2], as well as refining computational predictions, is essential. Experimentally, discovering new polymorphs is challenging because there is no set method. However, significant investments of time and money [3,4] can improve our understanding of polymorphic landscapes. For bioactive natural products (and their derivatives), controlling the solid state is important to preserve their biological properties.

**Scheme 1:**
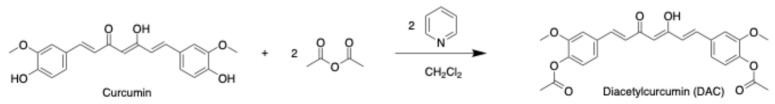
Synthesis of DAC.

Curcumin or diferuloylmethane [5,6], the natural major curcuminoid [7] extracted from the rhizome of *Curcuma longa* L., is a hydrophobic polyphenolic compound with well-documented biological activities such as antioxidant, anti-inflammatory, anticancer, cytotoxic and anti-Alzheimer’s disease effects [8,9]. Poor absorption, low bioavailability, and rapid metabolism [10] have hampered the clinical use of curcumin. Therefore, the search for polymorphs or the preparation of derivatives by replacing terminal phenol groups (-OH) has been a strategy for providing new curcuminoids that are more stable and soluble. When acetate groups take the place of phenols, diacetylcurcumin (DAC) is obtained [11,12] (see Scheme 1).

The results of various investigations have shown that DAC can bind to valuable vehicles such as proteins (bovine albumin and alpha-lactalbumin) [13,14], and, due to its lipophilicity, it has been inhibited in a murine model [15], thus demonstrating anti-arthritic activity. Furthermore, DAC has been shown to be effective at scavenging free radicals (e.g., NO and ·O_2_^−^) [16].

The acetyl groups in DAC are less reactive and facilitate permeability across biomembranes, which is important because DAC has the potential for use against resistant bacteria (*S. aureus*) [17]. In addition, when DAC has been tested in antimicrobial photodynamic therapy [18], it has been shown to exhibit greater antimicrobial activity than the parent molecule (curcumin).

A solid-state study of curcumin has led to the discovery of three polymorphs. In 1982, Hjorth Tonnesen et al. [19] reported the first crystal structure of curcumin (designated therein as Form 1, monoclinic, *P*2/*n*). Approximately 30 years later (2011), Sanphui et al. [20] reported two new polymorphs of curcumin in ChemComm, namely Form 2 (*Pca2*_1_) and Form 3 (*Pbca*), and found that the orthorhombic Form 2 exhibits better solubility.

Curcumin Form 1 was obtained from an ethanol (EtOH) solution by adding water as an anti-solvent. Polymorphs 2 and 3 were obtained using a protic polar solvent (EtOH) and the co-formers of crystallisation, 4-hydroxypyridine and 4,6-dihydroxy-5-nitropyrimidine, respectively. Other reports [21,22,23] have indicated that the three polymorphs can be obtained using chloroform, isopropanol, methanol and dimethyl sulfoxide.

In addition, several experimental methods have been developed to obtain curcumin polymorphs. A 1982 report demonstrated the use of slow evaporation and a cold solvent as the condition for nucleation of polymorph Form 1 [19]. Later, in 2011, polymorphs 2 and 3 were obtained using a different approach, in which mechanical kneading in a mortar prior to nucleation played a key role in the crystallisation process [20]. Furthermore, it has been reported that the polymorphs were obtained by supersaturating a curcumin solution at various temperatures. In a 2019 manuscript [24] on the relationships among curcumin polymorphs, it was noted that low pressure (100–400 mbar) affects intermolecular interactions, which facilitates nucleation. This feedback is important and shows that there is no single recipe for obtaining curcumin polymorphs, since knowledge-driven experimentation has been key to the different contributions.

DAC has been a promising derivative for the treatment of several human diseases, but the study of its polymorphic landscape has been lacking in the literature, and only a single crystal structure (monoclinic space group *P*2_1_, designated here as Form-1) has been reported in the past two decades (2004) [25].

Although there is no recipe that guarantees the discovery of polymorphs, herein we have performed a screen for DAC crystals. As a result, three polymorphs (two new ones) have been found and characterized by their single-crystal habits and by thermal analysis. To further characterize the non-equivalent carbons, solid-state attenuated total reflectance (ATR-IR) and Cross-Polarization Magic-Angle Spinning Nuclear Magnetic Resonance (CP-MAS-NMR) have been used. Finally, the total certainty of the DAC polymorphs has been established by single-crystal X-ray diffraction.

## 2. Results

Our approach for overall DAC polymorph detection was based on the following: we used several solvents with low, intermediate, and high dielectric constants. Solvents were chosen for their protic or aprotic nature. Experiments were performed at different temperatures, both room and cold, and under *vacuum* in some cases. The appearance of single crystals was visually inspected under an optical microscope. We rapidly analysed powders using ATR-IR spectroscopy for each batch. To authenticate polymorphs, we used ^13^C CP-MAS and X-ray diffraction techniques.

DAC was synthesised using acetic anhydride and pyridine as a catalyst (see previous reports) [26] and was characterised by liquid NMR (CDCl_3_), which showed the molecule as a fully enolised tautomer (enolic proton at 16 ppm), a key feature for the exercise of its important biological effects. Spectral characterisation, consistent with the literature [11,27], is included in the Supplementary Information (SI).

The obtention of DAC single crystals by slow evaporation of solvents under protection from light was carried out at room temperature or under refrigeration (2 °C). It is important to note that under these bottom-up experimental conditions, only two polymorphs were obtained (designated here as Form-1 and Form-3; see Table 1), which were first identified by their distinct crystalline forms under an optical microscope.

It was observed that the crystals that look like “fine bars” belong to Form-1, while those that look like “balloon shapes” correspond to Form-3. However, pure Form-1 (most abundant) was present in the majority of the common solvents screened, while the single crystal of Form-3 could only be identified in dichloromethane and acetone (where DAC is quite soluble). Although under the experimental conditions (see Table 1), the crystalline Form-2 (referred to as an elusive polymorph [28]) was not observed, the polymorphic Forms 1 (previously reported, CSD code EYIZER01 [29]) and 3 (concomitant polymorph [1]) were also obtained as a mixture of single crystals.

The experimental background [30] on obtaining polymorphs 2 and 3 of curcumin allowed us to establish the experimental conditions for the formation of crystalline powders, which were obtained immediately upon crystallisation by mixing solvents [20,31] and rapid cooling under vacuum. In this respect, n-hexane (*ε* = 1.89) or water (*ε* = 80) was used as an anti-solvent with immediate application of vacuum conditions (200 mbar). For the first attempt, polymorphic Form-2 was successfully obtained (see Table 2), while Form-1 was obtained in the subsequent five attempts. The polymorphic powders were authenticated using ATR-IR analysis, which showed that the carbonyl groups (CH_3_-C=O and C=O) exhibited subtle wavenumber differences among the polymorphs (Forms 1, 2, and 3).

**Figure 1:**
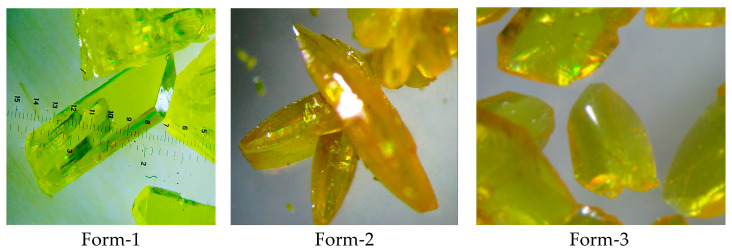
Views of DAC polymorphs under the microscope.

The single crystal of polymorph 2 was obtained using ethyl acetate and n-hexane (10%) by cooling the solvent mixture under vacuum (200 mbar) until nucleation was observed. The microscopic crystal patterns of the DAC polymorphs are highly distinctive and have been used to characterise each crystal; Figure 1 shows that the “fine bars”, “canoes”, and “balloon” shapes belong to Forms 1, 2, and 3, respectively.

DSC thermograms were obtained for all three polymorphs and are shown in the Supplementary Materials. Form-1 (m.p. = 173.89 °C) was found to have an intermediate melting point compared with Form-2 (m.p. = 164.66 °C) and Form-3 (m.p. = 175.56 °C).

ATR-IR proved an advantageous technique [32] for solid-state analysis of compounds, as only a minimal sample quantity is needed (on average, 1 mg) and no prior preparation is required. Furthermore, ATR-IR is a very economical technique for many determinations. In light of these advantages, DAC crystals and each batch obtained (see Table 1 and Table 2) were analysed by ATR-IR spectroscopy (see Supplementary Materials). Batch differentiation was primarily based on variations in the carbonyl group signals observed in ATR-IR spectra (see Table 3).

**Figure 2:**
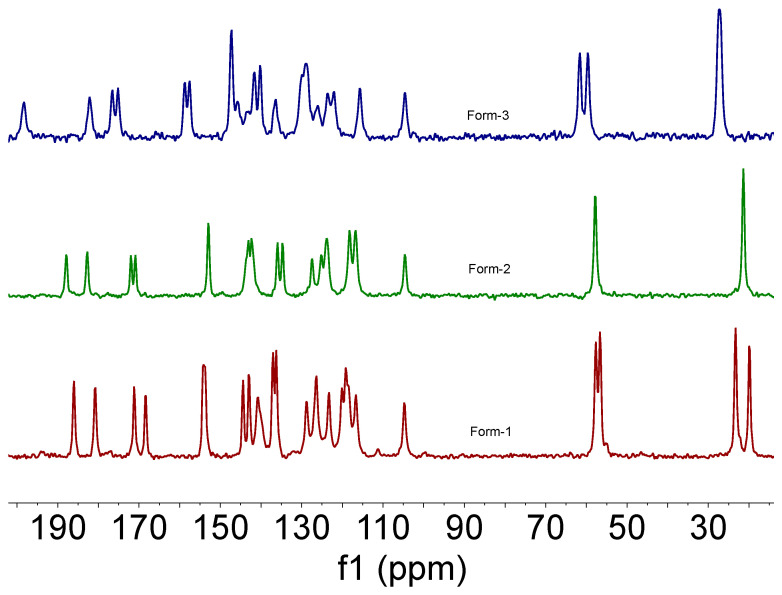
^13^C CP-MAS NMR spectra of DAC polymorphs.

Also, polymorph differentiation has been achieved using ^13^C CP-MAS NMR. Surprisingly, the non-equivalent carbons are observed as well-defined singlets, and no signal broadening is observed, as expected for crystalline materials [33], rather than amorphous materials (see Figure 2).

The three distinct crystal forms observed were authenticated by single-crystal X-ray diffraction, and full crystallographic data for the DAC polymorphs are provided in the Supplementary Materials and Table 4, indicating clear differences among the polymorphs.

The solubility of the polymorphs was measured in a water–ethanol (40%) mixture (see the Supplementary Materials), finding that polymorphic Form-2 (less stable) was more soluble than Form-3 (more stable), which agrees with the literature reporting an inverse relationship between stability and solubility [24].

## 3. Discussion

Diacetylcurcumin (DAC) was characterised by liquid ^1^H NMR in CDCl_3_. The hydrogen spectrum (see the Supplementary Materials) showed two singlets corresponding to the non-equivalent acetate (2.31 ppm) and methoxy groups (3.86 ppm) of the aromatic rings. Additionally, two double signals at 6.54 ppm and 7.60 ppm with *trans* coupling constants (15.8 Hz) were observed, corresponding to the vinyl groups of the heptanoid carbon chain. The central methine proton appears at 5.83 ppm, and aromatic protons between 7.04 ppm and 7.14 ppm. The keto–enol equilibrium was also observed [34]. The purity of the DAC was 99.6%, as determined by HPLC (see Supplementary Information), and the DART^+^ mass spectrum showed a peak at *m*/*z* 453 with 100% intensity (see Supplementary Materials), consistent with the molecular formula C_25_H_24_O_8_.

The differential scanning calorimetry (DSC) study revealed three distinct melting points for each DAC polymorph: Form-1 (173.89 °C), Form-2 (164.66 °C), and Form-3 (175.56 °C) (see the Supplementary Materials). While these data are consistent with the literature [11,15,16,35,36,37], the polymorphism of DAC has not been previously considered. The major features of the DSC diagrams suggest that polymorph Form-1 undergoes a minor rearrangement at 155.52 (ΔH = 17.48 J/g), while the main fusion occurs at 173.89 °C (ΔH = 113.94 J/g). Furthermore, polymorph Form-2 reveals a single major energy uptake (ΔH = 121.91 J/g) and melts at 164.66 °C. Polymorph Form-3 shows moderate energy uptake (ΔH = 27.06 J/g) and a clear fusion occurring at 175.56 °C. Interestingly, the second DSC trace reveals cold crystallisation, showing a new thermal fusion profile that closely resembles that of Form-2 [24]. A detailed X-ray powder diffraction study conducted at variable temperatures was not possible at present, but it may be a future step to further investigate the polymorphic relationships (enantiotropic or monotropic).

The structural characterisation of polymorphs by X-ray diffraction has been considered the gold standard for authenticating solid-state compounds. However, obtaining a single crystal can be problematic, so the issue of polymorphism is often overlooked. To address this, the ATR-IR technique [32] is particularly valuable for such a task. For example, Form-2 shows a signal at the lowest wavenumber (1752 cm^−1^) for the carbonyl groups of acetates (symmetric stretching), reflecting the high molecular symmetry of DAC. In contrast, Form-1 and Form-3 show a medium-intensity signal with the highest wavenumbers for the carbonyl group (C=O, corresponding to the keto–enol fraction, symmetric carbonyl stretching), while Form-2 displays a band with the lowest wavenumber.

The ATR-IR analysis has shown that the most distinctive bands correspond to the carbonyl groups of the acetate groups and were used to differentiate the three DAC polymorphs. This characteristic can be attributed to the strong interactions among acetate groups, as occurs in the crystal lattice of Form-2.

The DAC polymorphs were also analysed by solid-state NMR. Form-1 and Form-3 showed a greater number of signals characteristic of non-equivalent carbons (see Table 5), corresponding to methyl (-CH_3_), methoxy (-OCH_3_), acetate (-COO-), and carbonyl (C=O) groups, as described below. In contrast, Form-2 showed fewer signals because the molecule contains more symmetric carbons.

The differences in chemical shifts of ^13^C between the carbonyl groups (i.e., Δδ(^13^C) = δ(^13^C=O) − δ(^13^C=O)) revealed that polymorph 3 is the most unsymmetric compound (showing the largest difference Δδ(^13^C) = 16.16 ppm), followed by polymorphs 1 and 2. Furthermore, these data suggest that Form-2 exhibits an enolised proton (C=O···H···O=C) via resonance-assisted hydrogen bonding (RAHB) [38], which is consistent with the symmetry observed and confirmed by ATR-IR and X-ray analyses.

**Figure 3:**
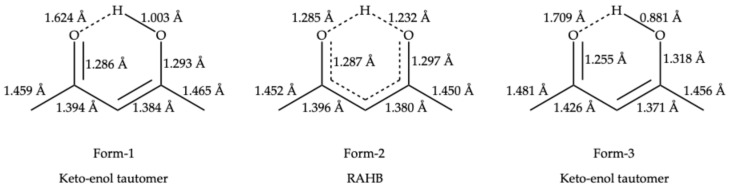
Bond distances of the keto–enol moiety of DAC polymorphs.

The keto–enol equilibria of DAC polymorphs were preserved in Form-1 and Form-3, whilst Form-2 showed a symmetric bond type via RAHB (C=O···H···O=C), which is depicted in Figure 3. This indicates that the chemical shift of the enolised proton would be approximately 17.5–18.0 ppm in ^1^H solid-state NMR, which agrees with a previous study [38]. The bond distances for the keto–enol moiety of the polymorphs were obtained from X-ray analysis, and the structural variations are clearly evident. Form-3 has an H···O bond length of less than 1 Å, which is more consistent with a keto–enol model.

Moreover, the differences in bond lengths between C-O and C=O are 1.318 − 1.255 = 0.063 Å for Form-3, 0.01 Å for Form-1, and 0.01 Å for Form-2, which are representative of unsymmetry; thus, a greater difference is expected for the more unsymmetric polymorph (Form-3).

**Figure 4:**
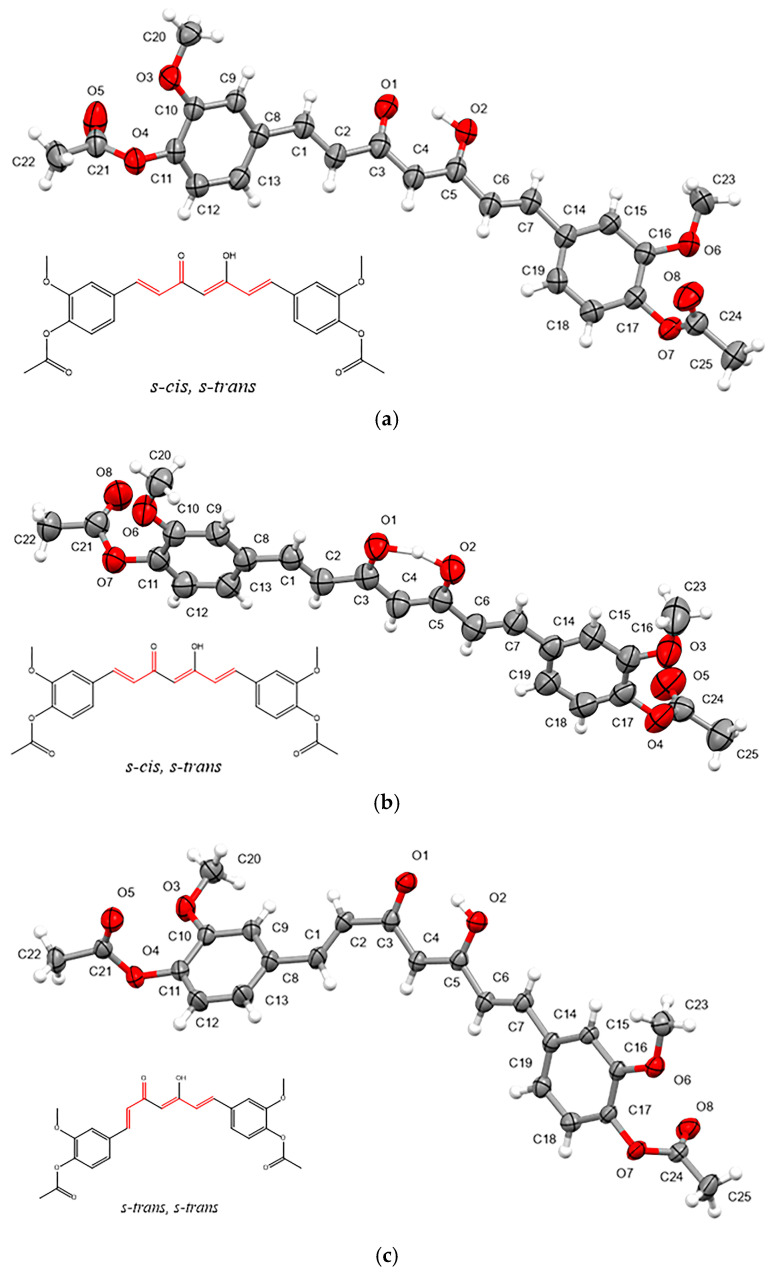
ORTEP diagram of diacetylcurcumin (**a**) Form-1, (**b**) Form-2 and (**c**) Form-3 at 50% probability of electron density for thermal ellipsoids.

The crystal structure of diacetylcurcumin (DAC) Form-1 was solved in monoclinic space group *P*2_1_ with one molecule in the asymmetric unit (Z’ = 1). The molecule exists in the β-keto–enol tautomer [((1*E*,3*Z*,6*E*)-5-hydroxy-3-oxohepta-1,3,6-triene-1,7-diyl) bis (2-methoxy-4,1-phenylene)diacetate] stabilized by a strong intramolecular hydrogen bond: O2−H2A ··· O1, 1.624(4) Å, (O2··· O1) 2.535(3) Å, (O2−H2A−O1) 148.61(4)°. Form-1 molecules adopt a fully extended linear structure with a curved, slightly twisted conformation. Phenyl rings at both extremes of the molecule are slightly twisted relative to each other, with both methoxy groups coplanar with the phenyl rings and oriented *syn* to the central keto−enol group. The acetate moieties are nearly perpendicular to the phenyl rings (71.50 and 75.68°) and oriented to opposite sides of the molecule. Weak C−H···O interactions, (C15−H15···O8): 2.40 Å, 3.280(4) Å, 157.5°; C20−H20C···O5: 2.66 Å, 3.486(5) Å, 144.6° and C22−H22C···O7: 2.64 Å, 3.500(4) Å, 150.0°, also play an important role in the overall molecular aggregation. Several samples (though not exhaustively) were examined, and, in all cases, the report in CSD CODE: EYIZER01 [29] shows the same absolute structure as in Figure 4a.

The crystal structure of DAC Form-2 was solved in the monoclinic space group *P*2_1_/c with one molecule in the asymmetric unit (Z’ = 1). As found for Form-1, the molecule exists also in the β-keto–enol tautomer [((1*E*,3*Z*,6*E*)-5-hydroxy-3-oxohepta-1,3,6-triene-1,7-diyl) bis (2-methoxy-4,1-phenylene)diacetate] with an even stronger intramolecular hydrogen bond: O2···H2A···O1, 1.285 and 1.232(3) Å, (O2···O1) 2.466(2) Å, (O2−H2A−O1) 156.89(3)°. In contrast with the Form-1 molecule, the overall conformation is nearly planar with the acetate moieties almost perpendicular to the phenyl rings (76.64 and 82.31°) but now oriented toward the same side of the molecule (Figure 4b). There are C−H···O intermolecular interactions from methoxy and the acetoxy groups of one of the phenyl rings (C20−H20B···O2: 2.57 Å, 3.243(3) Å, 127.6°, C22−H22B···O5: 2.46 Å, 3.416 (4) Å, 172.6° and C22−H22C···O1: 2.49 Å, 3.433(3) Å, 166.0°) in addition to the C−H···O interaction, C4−H4···O5: 2.48 Å, 3.342(3) Å, 153.8°, from olefinic hydrogens.

The Form-3 crystal structure was solved in the triclinic space group *P*−1 and with one molecule in the asymmetric unit. A strong intramolecular hydrogen bond, O2−H2A···O1: 1.709(3) Å, (O2···O1) 2.518(17) Å, (O2−H2A−O1) 151.42(3)°, is present in the enol tautomer [((1*E*,3*Z*,6*E*)-5-hydroxy-3-oxohepta-1,3,6-triene-1,7-diyl) bis (2-methoxy-4,1-phenylene)diacetate]. In contrast to Form-1 and Form-2, molecules of Form-3 adopt a folded L-type conformation. The orientation of the acetate moieties is again almost perpendicular to the phenyl rings (77.32 and 80.73°) but oriented towards the opposite side of the molecule, as found in Form-1 (Figure 4c). There are C−H···O intermolecular interactions involving the methoxy and acetoxy groups of one of the phenyl rings. The following interactions were observed: C20−H20C···O5: 2.63 Å, 3.407(2) Å, 138.9°; C22−H22A···O1: 2.54 Å, 3.494(2) Å, 173.1°. Three more interactions involve olefinic and aromatic hydrogens: C2−H2···O5: 2.59 Å, 3.515(2) Å, 172.5°; C9−H9···O5: 2.60 Å, 3.494(2) Å, 146.2°; and C15−H15···O8: 2.47 Å, 3.305(2) Å, 149.8°.

Intermolecular interactions such as C−H···O are weaker in the hydrogen-bond hierarchy and are of secondary importance in directing supramolecular assembly. However, in the absence of strong hydrogen bonds, weaker interactions can be the major determinants of the overall crystal packing.

**Figure 5:**
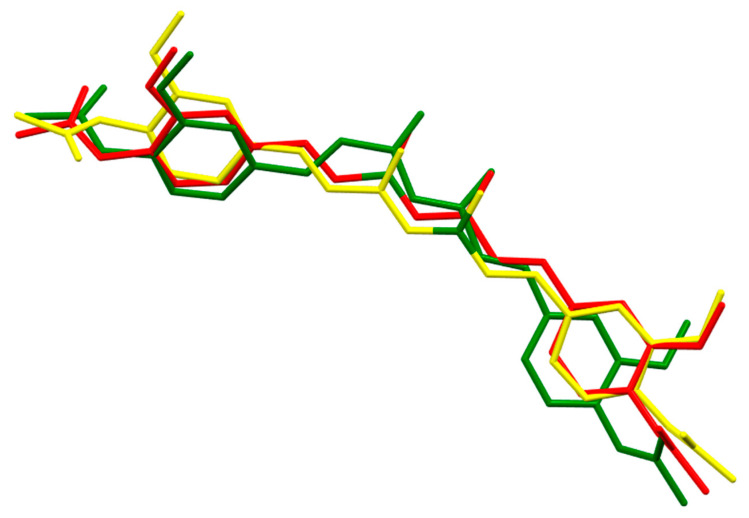
Molecular overlay of diacetylcurcumin polymorphs. Colour code: Form-1, red; Form-2, yellow; and Form-3, green.

Torsional flexibility inside the polymorphs suggests that diacetylcurcumin exists as conformational polymorphs [39,40] (Table 6). Conformation comparisons across the three polymorphs of diacetylcurcumin were calculated using the TORMAT program [41] (RMSD; 1 vs. 2: 0.880591, 1 vs. 3: 1.4987, 2 vs. 3: 1.28418) and are displayed in Figure 5. Based on the orientation of −OCH_3_ groups with respect to the central keto−enol group, these conformers can be described as *syn*–*syn*. A notable feature of Form-3 is the arrangement of its C1−C2−C3−O1 fragment (Figure 4c), with C1 oriented *trans* to O1 (*s*-*trans* configuration) contrary to the observed *s*-*cis* configurations of Forms 1 and 2. In all structures, the C4−C5−C6−C7 fragment is nearly planar and exhibits an all-*trans* (*s*-*trans*) arrangement, as shown in Figure 4 and Table 6. In addition to the high degree of planarity, there is a notable absence of π··π stacking between molecules, as confirmed using the Aromatics Analyser component of the Mercury program; the separation between the ring centroids exceeds the range of a phenyl···phenyl interaction in all cases (Minimal Distance between ring centroids; Form-1: 6.138(14) Å, Form-2: 4.7463(16) Å; Form-3: 5.0720(11) Å).

The XPac geometrical analysis [42] of the three polymorphic forms shows that there is no isostructurality; only 1D dimensionality of the common packing fragment is observed between Form-1 and Form-3 (see the Supplementary Material).

**Figure 6:**
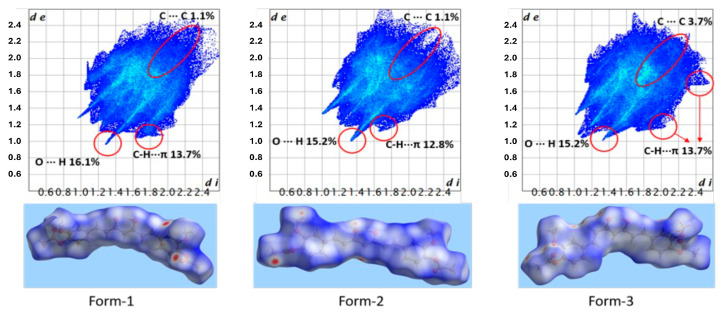
Principal contributions of intermolecular interactions in the Hirshfeld surface area are highlighted in red circles for Form-1 to Form-3. The percentage of O···H and C-H···and C···C interactions for molecules is indicated, and the remaining blue area indicates interactions that belong to H···H contacts.

Similarities and differences among the diacetylcurcumin polymorphs in the crystalline forms (Forms 1, 2, and 3) have been elucidated using Hirshfeld surface analysis, 2D fingerprinting, and intermolecular interaction analysis with the CrystalExplorer program [43]. The surface analysis diagrams of the three crystal forms are shown in Figure 6.

The 2D fingerprint of intermolecular interactions indicates that the percentage contributions of C-H···π and O···H contacts are similar. Among the three crystal forms, only the carbonyl oxygen atoms on the acetoxy group and the central keto−enol groups of DAC act as hydrogen-bond acceptors, forming O···H−C interactions, which are clearly visible as spikes. In addition to the large portions of planar structures, notably the absence of π···π interactions, the H···H, C···H, and O···H forces are the main components (~70%) of the entire Hirshfeld surface.

**Figure 7:**
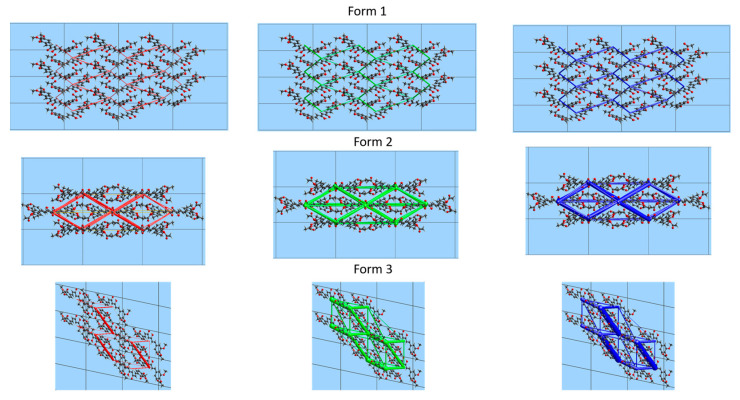
Energy framework diagrams viewed along the *a*-axis, with an energy scale factor of 100. Red: Coulomb energy; green: dispersion energy and blue: total energy.

The crystal packing in the polymorphs is constructed through the ‘energy frameworks’ method integrated in CrystalExplorer. All three DAC polymorphic forms show differences in the strength of interactions between the pair of molecules involved (Figure 7), and in all cases, the dispersion energy framework is dominant over the electrostatic energy framework, playing an important role in crystal packing arrangements, while their total interaction energies correlate well with their relative stability.

**Figure 8:**
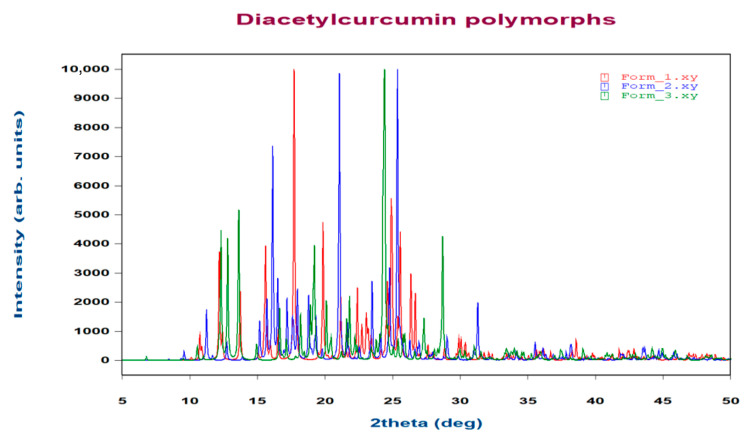
Simulated PXRD powder X-ray diffraction patterns for the three polymorphs, using CuKα wavelength radiation.

The intermolecular energy calculations were performed with the CLP-PIXEL program [44] (Table 7) and show that the total energies of Form-1, Form-2, and Form-3 agree well with the thermal stability indicated by the melting point. The energy calculations for DAC polymorphs indicate that the most stable polymorph has a lower free energy [45], indicating that the most stable crystallographic structure of diacetylcurcumin is Form-3 (262.2 kJ mol^−1^), Form-1 is metastable (247.6 kJ mol^−1^), and the most unstable is Form-2 (245.4 kJ mol^−1^). Figure 8 shows the overlaid calculated powder patterns for the three polymorphs. The independent traces generated using Mercury software (v2024.3.0) were derived from the well-defined .cif archives and are available in the Supplementary Materials.

It is surprising that Form-3 (considered the most stable) showed the lowest probability of crystallisation in the single-crystal formation tests by slow evaporation of a single solvent. This result correlates well with Ostwald’s rule [46,47], as a low supersaturation rate allows sufficient time for a solute to reorganise and produce the growth of a more stable crystalline form.

Form-3 was obtained by slow evaporation under refrigeration, using dichloromethane (10 days) and acetone (5 days) as solvents. This contrasts with crystallisation at room temperature (shorter evaporation time), which yields crystalline Form-1.

At a rapid supersaturation rate [48], kinetic effects predominate, explaining the discovery of the elusive polymorph (Form-2). In the crystallisation experiment with the solvent mixture using ethyl acetate and hexane (lower boiling point) under *vacuum*, high cooling or evaporation rates also occurred, resulting in a less stable polymorph (Form-2). Conversely, at a low supersaturation rate, thermodynamic effects prevail, which are suitable for the formation of the more stable Form-3.

The solubility of the three DAC polymorphs was determined in a solvent mixture of ethanol/water (4/6). Although DAC is very insoluble in this medium, a direct correlation was found between solubility and stability. Form-2 is the most soluble (2.29 × 10^−5^ µM); the metastable Form-1 has intermediate solubility (1.20 × 10^−5^ µM); and Form-3 (1.16 × 10^−5^ µM) is the least soluble. Therefore, the stability of these polymorphs follows the order: Form-3 > Form-1 > Form-2.

## 4. Materials and Methods

All solvents and reagents were purchased from Sigma-Aldrich (Sigma-Aldrich S. de R.L de C.V., Toluca, México) and used without further purification.

Five grams (13.57 mmol) of curcumin was dissolved in 100 mL of dichloromethane. Subsequently, 2.20 mL (27.14 mmol) of pyridine was added, followed by the addition of 2.2 equivalents of acetic anhydride (2.75 mL, 29.84 mmol). The reaction mixture was stirred magnetically overnight at room temperature. The solvent was then evaporated, and the residue was extracted with ethyl acetate (3 × 60 mL) and water. The organic phase was dried over anhydrous Na_2_SO_4_ and evaporated under reduced pressure. A yellow solid was obtained in 80% yield.

Approximately 100 mg of DAC powder was dissolved in 15 mL of a single solvent (with different dielectric constants [49]). These solutions were kept in slow evaporation at room temperature and protected from light. Once the appropriate crystalline medium was observed, the product was filtered through a Hirsch funnel and characterised by ATR-IR. The same procedure was applied for slow cold evaporation, except that a refrigerator set to 2 °C was used.

Approximately 100 mg of DAC powder was dissolved in 15 mL of a solvent (with different dielectric constants). Then, 10% of the corresponding anti-solvent (n-hexane or water) was added to each solution. These solutions were placed in a vacuum chamber (200 mbar) at room temperature, and rapid supersaturation of the medium was observed. Finally, the resulting product was filtered using a Hirsch funnel and characterised by ATR-IR spectroscopy.

Liquid spectra (^1^H-NMR, ^13^C-NMR), and two-dimensional spectra (HSQC and HMBC, see the Supplementary Materials) were recorded on a Bruker Avance III 400 MHz spectrometer (Bruker, Rheinstetten, Germany) using CDCl_3_ as a solvent and TMS as a reference; all spectra were processed using the MNova program (v17.0.0) [50].

HPLC (see Supplementary Materials) for DAC was performed as indicated in the literature [51], with minor modifications. Chromatograms were recorded using Agilent 1200 equipment (Santa Clara, CA, USA) with a UV diode-array detector (see UV spectrum for DAC in Supplementary Materials) and a Waters-2996 detector at 410 nm, using an isocratic solvent from acetonitrile/water 55:45 (orthophosphoric acid 0.02%), a flow rate of 1 mL/min, and a Column Spherisorb (Waters Corp., Milford, MA, USA) 5 mm ODS1 250 X 4.6 mm as the stationary phase.

Infrared spectra were recorded on an FT-IR NICOLET IS-50 (Thermo Fisher Scien-tific, Waltham, MA, USA) using the attenuated total reflectance (ATR) technique (4000–400 cm−1).

The mass spectrum was recorded using the AccuTOF JMS-T100LC JEOL (JEOL de Mexico SA de CV, Mexico City, Mexico) equipment for DART^+^, at 350 °C, in positive ion mode and calibration was standard with PEG 600 [52]. The mass spectrum is shown in the Supplementary Material.

TGA and DSC analysis were carried out with a thermobalance TA Instruments, model Discovery 250 (Waters Corp., New Castle, DE, USA), using an aluminium crucible and outer bottom Ø 5 mm. The sample was heated from 25 °C to 200 °C at a heating rate of 10 °C min^−1^ under a nitrogen atmosphere [53].

The ^13^C CPMAS-NMR spectra were recorded using a Bruker 500 MHz spectrometer (Bruker, Mexico City, México) equipped with a probe PH MAS DVT (N-P/F/H).

Crystal structure and refinement details. Intensity data were collected using a Bruker Apex diffractometer (Bruker Corp., Billerica, MA, USA) for Form-2 and Form-3 and a Bruker D8 Venture κ geometry diffractometer (Bruker, Karlsruhe, Germany) for Form-1. An absorption correction based on equivalent reflections was applied to the data. The structures were solved with the ShelXS (v2019/3) [54] program and refined with ShelXL (v2014/2) [55] managed in SHELXLe (v2011) [56]. The models were refined using full-matrix least-squares minimisation on *F*^2^. Atomic hydrogen positions for those bonded to O atoms were located on a difference electron-density map at an advanced refinement stage, while hydrogen atomic positions for those bonded to C atoms were calculated from assumed geometries. Hydrogen atoms were included in structure factor calculations, but they were not refined. Isotropic displacement parameters of all the hydrogen atoms were approximated from the U(eq) value of the atom they were bonded to. The checkCIF/PLATON program (v2015) was used to validate the structures, and the publication materials were prepared with CIFTAB and Mercury (v2025.1.1) [57].

Solubility studies were performed in an ethanol–water (4/6) mixture [20]. Then, 5 mg of polymorphs 1, 2, or 3 was weighed and suspended in 5 mL of the solvent mixture. The suspension was kept under constant stirring for 24 h. Subsequently, a 1 mL aliquot was taken and filtered through a 4 µm sintered filtration flask. From the filtrate, 100 microliters were taken and measured in triplicate at 410 nm in an ELISA microwell plate. The absorbance was then interpolated on the DAC standard curve in ethanol (see Supplementary Materials) to determine the micromolar concentration of each polymorph.

## 5. Conclusions

The polymorphic landscape of solid compounds can be explored through trial and error, though serendipity sometimes plays a role. Here, we rationally selected solvents and quickly analysed the resulting solids using ATR-IR. This approach reduced both time and money spent on analysis. Form-2 (unstable) was obtained in solvents with the lowest dielectric constants, such as ethyl acetate/n-hexane, and was rapidly cooled under vacuum. Form-3 (more stable) was found in solvents with intermediate dielectric constants and longer crystallisation times. Form-1 (metastable) does not depend on a particular dielectric constant and appears in most solvents and conditions.

We did not use any crystallisation co-formers, thereby ruling out the presence of a co-crystallisation molecule. This study provides a complementary examination of acetylated curcumin polymorphs using ATR-IR, which successfully helped identify each polymorphic species. We encounter well-defined technical and scientific challenges when searching for and characterising curcuminoid polymorphs, especially given the growing therapeutic interest and the potential to achieve optimal bioavailability. We expect this experimental approach will benefit the discovery of new polymorphic landscapes in other natural or synthetic compounds.

## Figures and Tables

**Table 1:** Screening of solvents for the obtention of crystals of DAC.

Solvent	*ε* ^a^	Temperature	Time (Days)	Polymorph Observed
Ethyl acetate	6.02	room	3	Form-1
Ethyl acetate	6.02	refrigerator	5	Form-1 and Form-3
Dichloromethane	8.93	room	5	Form-1
Dichloromethane	8.93	refrigerator	10	Form-3
Acetone	20.7	room	2	Form-1
Acetone	20.7	refrigerator	5	Form-3
Ethanol	24.5	room	1	Form-1
Ethanol	24.5	refrigerator	1	Form-1
Methanol	32.7	room	1	Form-1 and Form-3
Methanol	32.7	refrigerator	1	Form-1
Acetonitrile	37.5	room	1	Form-1
Acetonitrile	37.5	refrigerator	3	Form-1 and Form-3

^a^ Dielectric constant.

**Table 2:** Immediate nucleation of DAC under solvent mixtures with the use of vacuum (200 mbar).

Attempt	Solvent	Anti-Solvent	Polymorph
1	Ethyl acetate	Hexane	Form-2
2	Dichloromethane	Hexane	Form-1
3	Acetone	Hexane	Form-1
4	Ethanol	Water	Form-1
5	Methanol	Water	Form-1
6	Acetonitrile	Water	Form-1

**Table 3:** Representative signals of carbonyl groups of DAC polymorphs by ATR-IR.

Carbonyl Group (C=O)	Form-1 cm^−1^ (Intensity)	Form-2 cm^−1^ (Intensity)	Form-3 cm^−1^ (Intensity)
Acetate moiety	1756 (strong)	1752 (strong)	1762 (strong)
Keto moiety	1629 (medium)	1625 (medium)	1633 (medium)

**Table 4:** Crystal parameters of DAC polymorphs ^a^.

Property	Form-1	Form-2	Form-3
Space group	*P*2_1_	*P*2_1_/n	*P*−1
a/Å	8.8478(2)	11.4469(19)	9.1473(2)
b/Å	7.9351(2)	10.9829(18)	10.3385(3)
c/Å	16.4135(4)	18.728(3)	13.2791(3)
α/°	90.00	90.00	78.850(1)
β/°	98.564(1)	99.863(3)	89.336(1)
γ/°	90.00	90.00	66.556(1)
Volume/Å^3^	1139.51(5)	2319.7(7)	1127.4(1)
*D*_calcd_Mg m^−3^	1.319	1.296	1.333
Z	2	4	2
*R*_1_ [*I* > 2σ(*I*)]	0.0402	0.0539	0.0564
*wR*_2_ (all)	0.1024	0.1528	0.1580
Goodness-of-fit	1.079	1.014	1.027

^a^ Full crystal data is given in Supplementary Materials.

**Table 5:** ^13^C CPMAS of DAC polymorphs, chemical shifts in ppm.

^13^C	Form-1	Form-2	Form-3
-CH_3_	23.31, 19.89	21.38	27.27
-OCH_3_	57.65, 56.63	57.79	61.61, 59.61
-COO	171.17, 168.37	172.00, 170.88	176.57, 175.18
-C=O	186, 180.79	187.87, 182.74	198.30, 182.14

**Table 6:** Selected torsion angles [°] for Forms 1–3.

Atoms	Form-1	Form-2	Form-3
C(8)-C(1)-C(2)-C(3)	−179.8(3)	−179.9(2)	176.22(18)
C(1)-C(2)-C(3)-O(1)	−14.4(5)	−2.4(4)	−151.80(19)
C(1)-C(2)-C(3)-C(4)	166.0(3)	176.8(2)	27.5(3)
C(2)-C(3)-C(4)-C(5)	178.7(3)	179.5(2)	−174.89(15)
C(3)-C(4)-C(5)-C(6)	−178.1(3)	−179.6(2)	175.47(15)
O(2)-C(5)-C(6)-C(7)	−3.7(5)	−2.8(4)	4.3(3)
C(4)-C(5)-C(6)-C(7)	176.0(3)	177.9(2)	−173.84(16)
C(5)-C(6)-C(7)-C(14)	−180.0(3)	−179.2(2)	176.61(15)

**Table 7:** Breakdown of interaction energies (kJ mol^−1^) into Coulombic, polarization, dispersion, dipolar and repulsion components for the polymorphs of diacetylcurcumin.

Polymorph	E_coul_	E_pol_	E_dis_	Er_ep_	E_dip_	E_tot_
Form-1	−35.5	−72.1	−205.5	67.1	−1.7	−247.6
Form-2	−43.9	−70.8	−196.8	66.1	-----	−245.4
Form-3	−46.0	−77.5	−214.5	75.8	-----	−262.2

## Data Availability

The data supporting this research can be obtained from the Supplementary Information (SI).

## Supplementary Materials

The following supporting information can be downloaded at: https://www.mdpi.com/article/10.3390/molecules31122133/s1. The crystallographic data for Form-1, Form-2 and Form-3 were deposited (including fcf files) in the Cambridge Crystallographic Data Centre (CCDC) with the Numbers 2490425 (Form-1), 2490426 (Form-2), 2490427 (Form-3). Table S1 shows the crystal data and structure refinement of DAC polymorphs.
