# Supplementary material for: The Polymorphs of Diacetylcurcumin (DAC)
Source: Molecules. 2026 Jun 17;31(12):2133. doi: 10.3390/molecules31122133 (PMC13305184; doi:10.3390/molecules31122133)
Supplement: Supplementary file 1 [file molecules-31-02133-s001.zip › molecules-4349143-supplementary.pdf]

Supporting Information

The Polymorphs of Diacetylcurcumin (DAC)

Marco A. Obregón-Mendoza [1], Rosario Tavera-Hernández [1], Rubén Sánchez-Obregón [1], Carolina Escobedo-Martínez [2] , Rubén A. Toscano [1] and Raúl G. Enríquez [1,*]

Index

Figure S1. ^{1}H-NMR spectrum of DAC (400 MHz, $CDCl_3$). 3
Figure S2. ^{1}H-NMR spectrum of DAC (aromatic region expantion, 400 MHz, $CDCl_3$). 3
Figure S3. ^{13}C-NMR spectrum of DAC (100 MHz, $CDCl_3$). 4
Figure S4. HSQC-spectrum of DAC ($CDCl_3$). 4
Figure S5. HMBC-spectrum of DAC ($CDCl_3$). 5
Figure S6. HPLC chromatogram of DAC purity 99.61 %. 5
Figure S7. UV-spectrum of DAC 6
Figure S8. Mass spectrometry of DAC, DART^{+}, molecular ion peak at 453^{+}. 6
Figure S9. DSC of DAC Form-1 7
Figure S10. DSC of DAC Form-2 7
Figure S11. DSC of DAC Form-3 7
Figure S12. IR-ATR spectrum DAC (crystallization of ethyl acetate) at room temperature. 8
Figure S13. IR-ATR spectrum DAC (crystallization of ethyl acetate) at 2 °C. 8
Figure S14. IR-ATR spectrum DAC (crystallization of dichloromethane) at room temperature. 9
Figure S15. IR-ATR spectrum DAC (crystallization of dichloromethane) at 2 °C. 9
Figure S16. IR-ATR spectrum DAC (crystallization of acetone) at room temperature 10
Figure S17. IR-ATR spectrum DAC (crystallization of acetone) at 2 °C. 10
Figure S18. IR-ATR spectrum DAC (crystallization of ethanol) at room temperature. 11
Figure S19. IR-ATR spectrum DAC (crystallization of ethanol) at 2 °C. 11
Figure S20. IR-ATR spectrum DAC (crystallization of methanol) at room temperature. 12
Figure S21. IR-ATR spectrum DAC (crystallization of methanol) at 2 °C. 12
Figure S22. IR-ATR spectrum DAC (crystallization of acetonitrile) at room temperature. 13
Figure S23. IR-ATR spectrum DAC (crystallization of acetonitrile) at 2 °C. 13
Figure S24. IR-ATR spectrum DAC (crystallization of ethyl acetate and hexane). 14
Figure S25. IR-ATR spectrum DAC (crystallization of dichlromethane and hexane). 14
Figure S26. IR-ATR spectrum DAC (crystallization of acetone and hexane). 15
Figure S27. IR-ATR spectrum DAC (crystallization of ethanol and water). 15
Figure S28. IR-ATR spectrum DAC (crystallization of methanol and water). 16
Figure S29. IR-ATR spectrum DAC (crystallization of acetonitrile and water). 16
Figure S30. XPac windows for the comparison of the structure pairs Form 1–Form 2, Form 1–Form 3 and Form 2–Form 3. 17
Table S1. Crystal data and structure refinement of DAC polymorphs 18
Figure S31. Curve standard of DAC in ethanol. 19
Figure S32. Views of DAC polymorphs (under microscope) and size crystals obtained by X-Ray. 20
Figure S33. Simulated PXRD powder X-ray diffraction patterns for the three polymorphs. 21

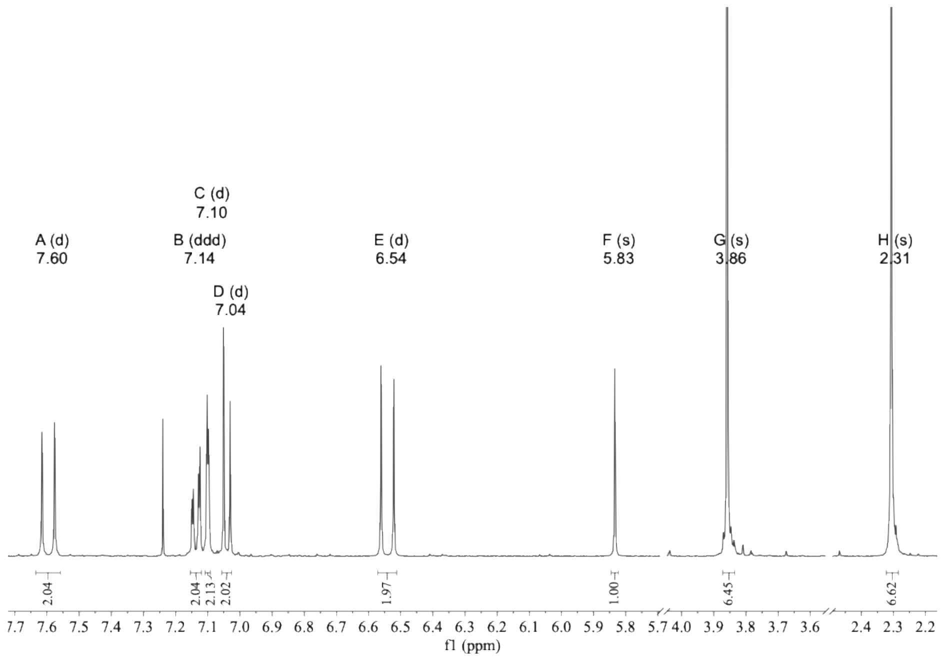

Figure S1. ^{1}H-NMR spectrum of DAC (400 MHz, $CDCl_3$).

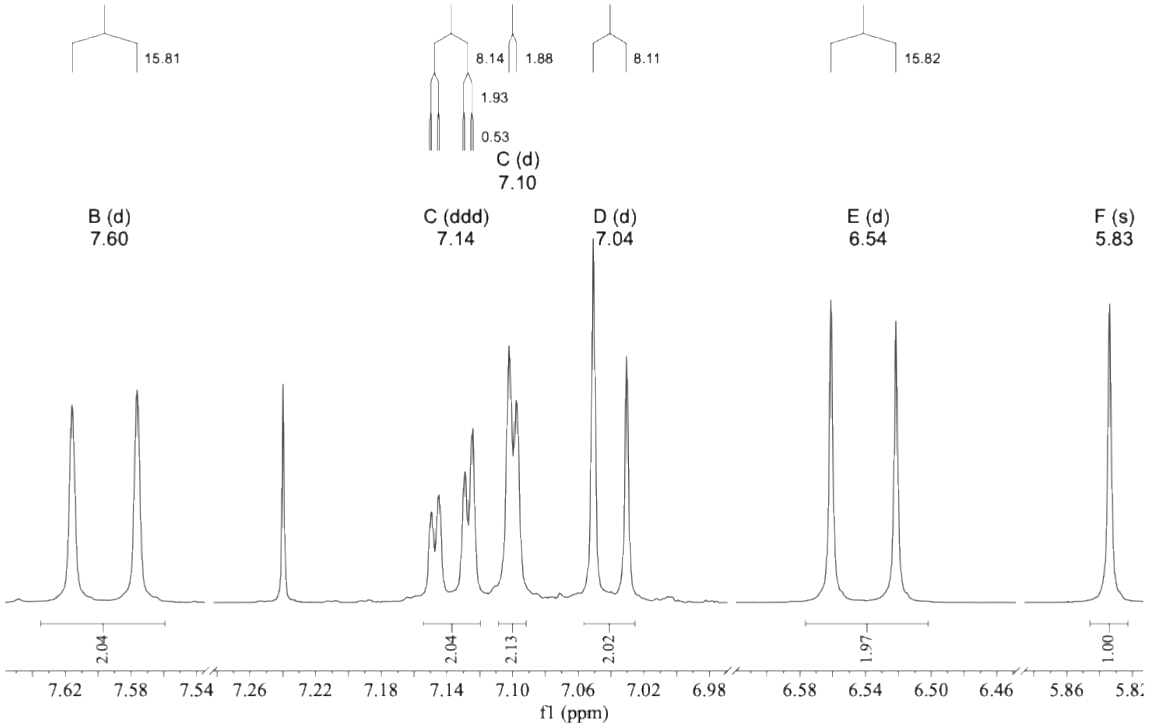

Figure S2. ^{1}H-NMR spectrum of DAC (aromatic region expantion, 400 MHz, $CDCl_3$).

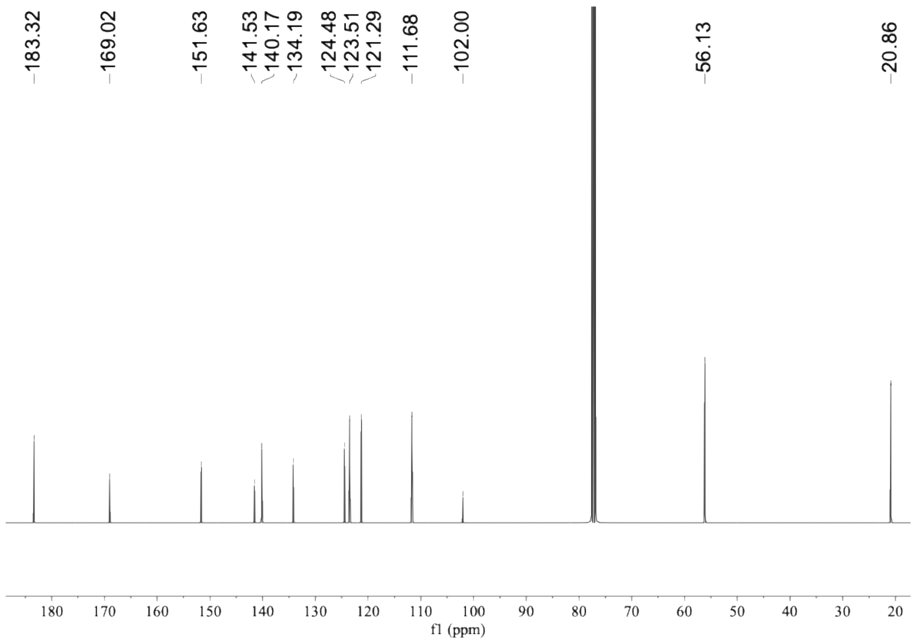

Figure S3. ^{13}C-NMR spectrum of DAC (100 MHz, $CDCl_3$).

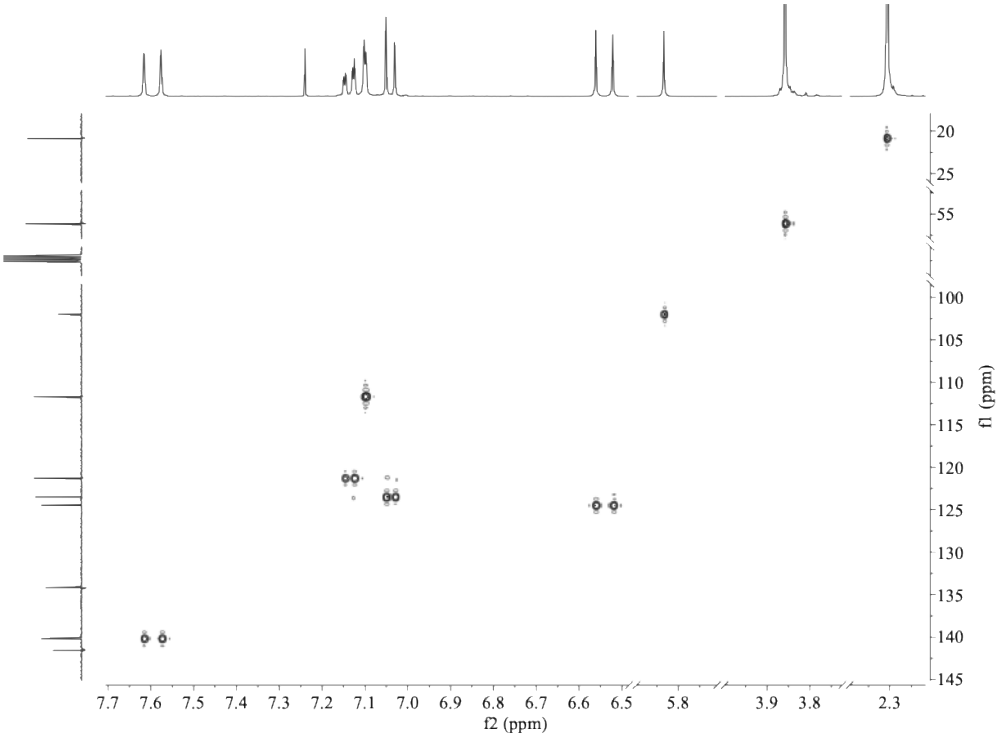

Figure S4. HSQC-spectrum of DAC ($CDCl_3$).

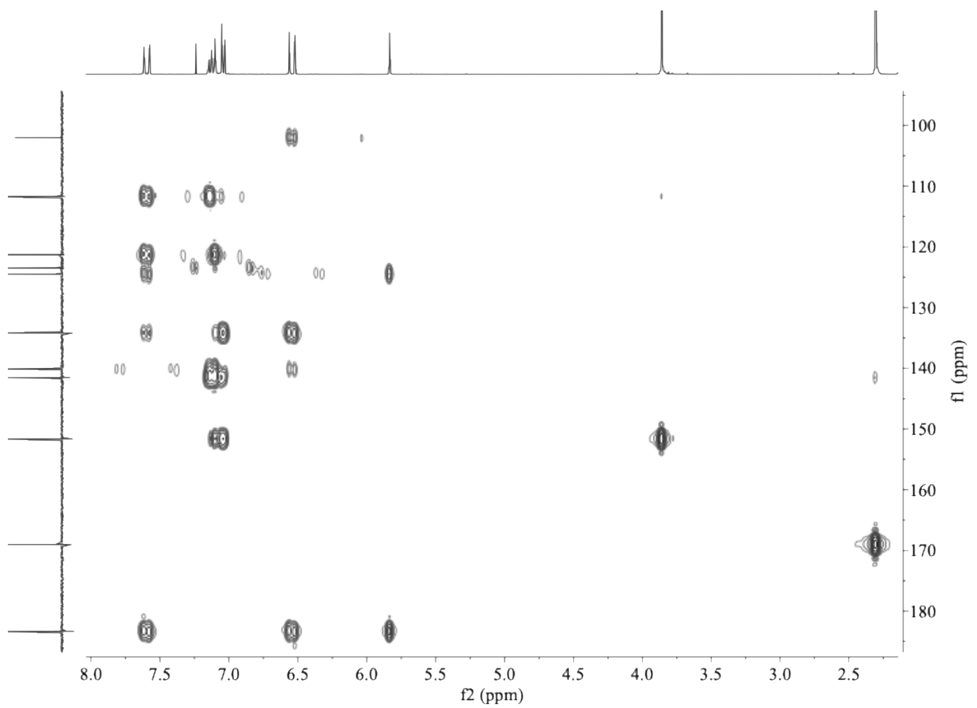

Figure S5. HMBC-spectrum of DAC ($CDCl_3$).

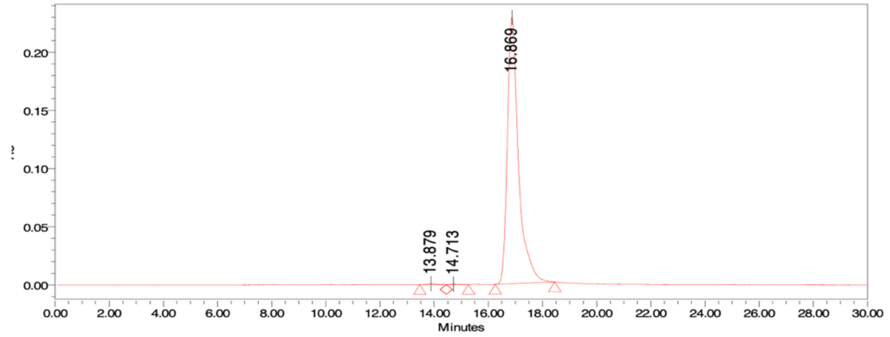

	Nombre	RT	Area	% Area
1		13.879	19927	0.30
2		14.713	5674	0.09
3		16.869	6567177	99.61

Figure S6. HPLC chromatogram of DAC purity 99.61 %. Column Spherisorb 5 mm ODS1 250 X 4.6 mm, Mixture of solvents acetonitrile (55 %) water (45 % and 0.02 % orthophosphoric acid), flow 1 mL / minute.

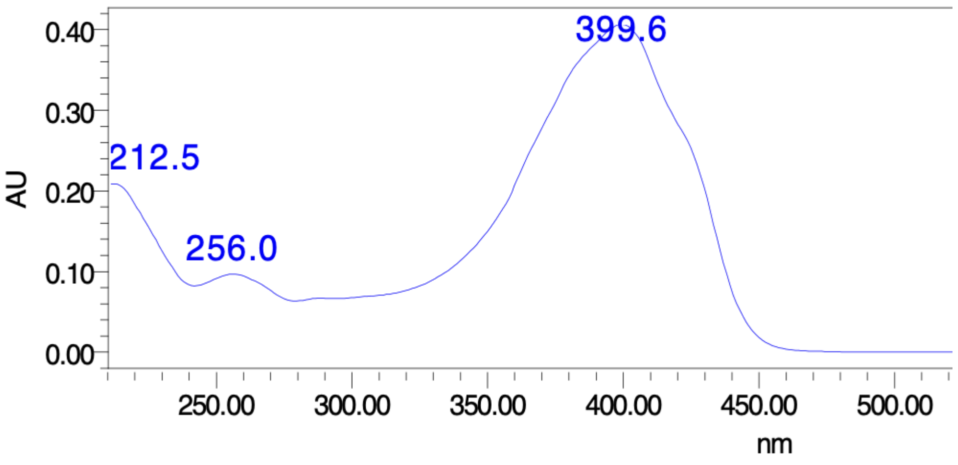

Figure S7. UV-spectrum of DAC

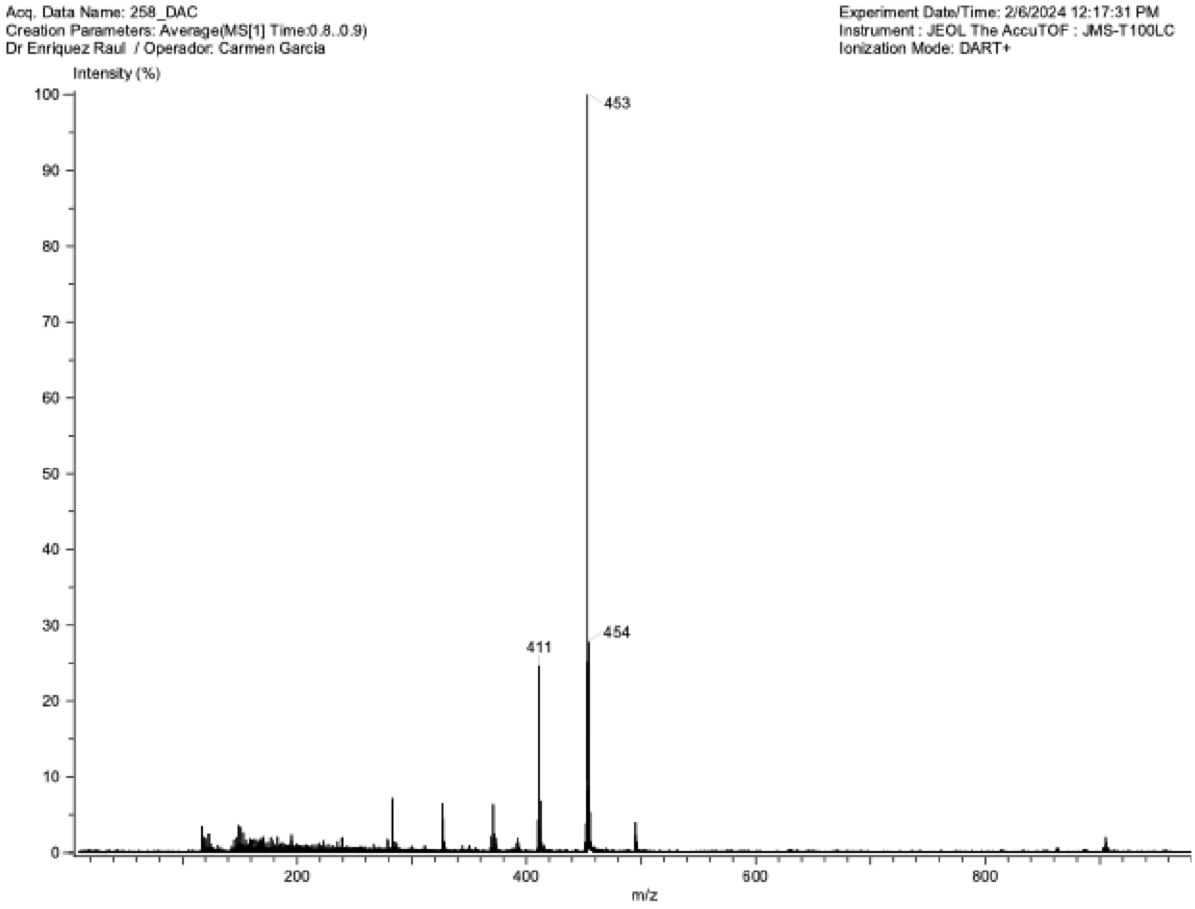

Figure S8. Mass spectrometry of DAC, DART$^+$, molecular ion peak at 453$^+$.

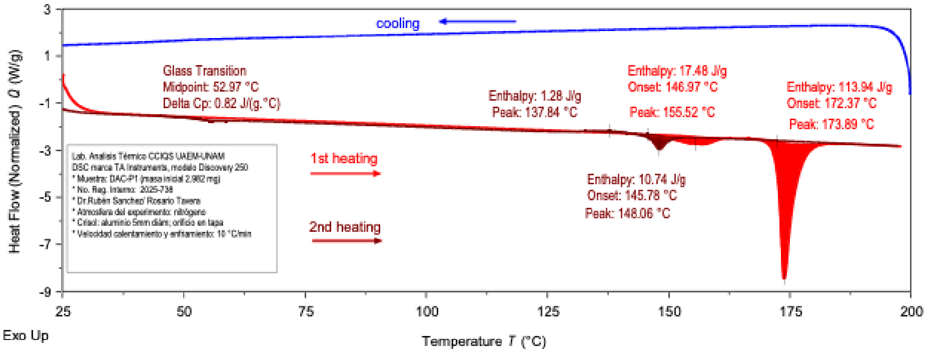

Figure S9. DSC of DAC Form-1

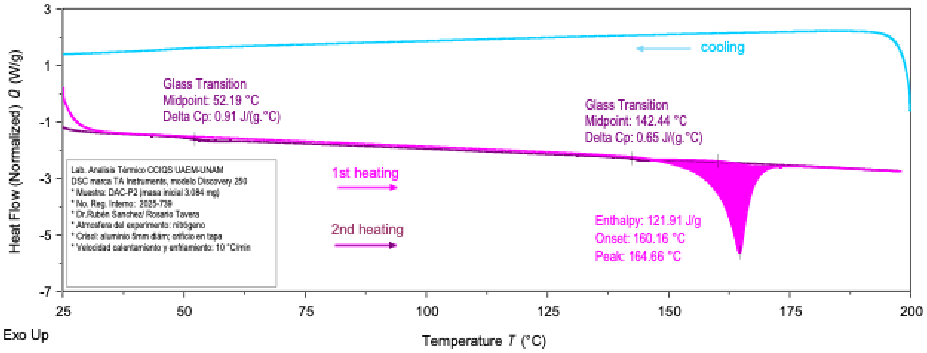

Figure S10. DSC of DAC Form-2

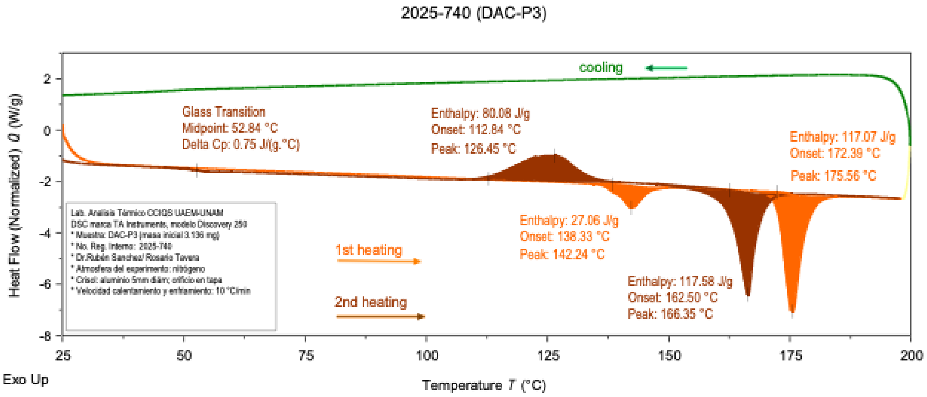

Figure S11. DSC of DAC Form-3

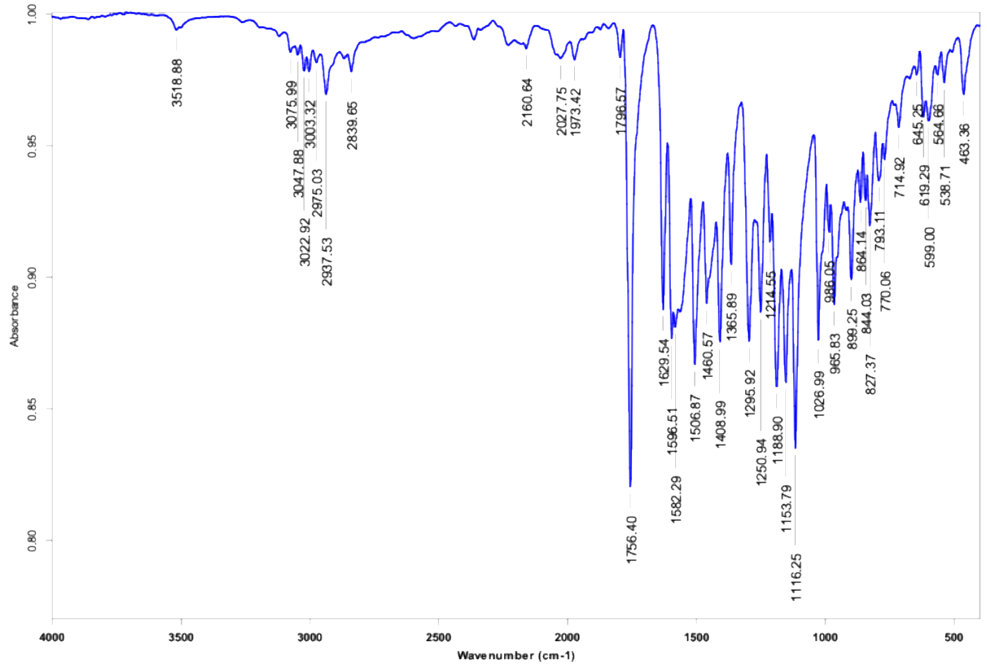

Figure S12. IR-ATR spectrum DAC (crystallization of ethyl acetate) at room temperature. Corresponds to Form-1.

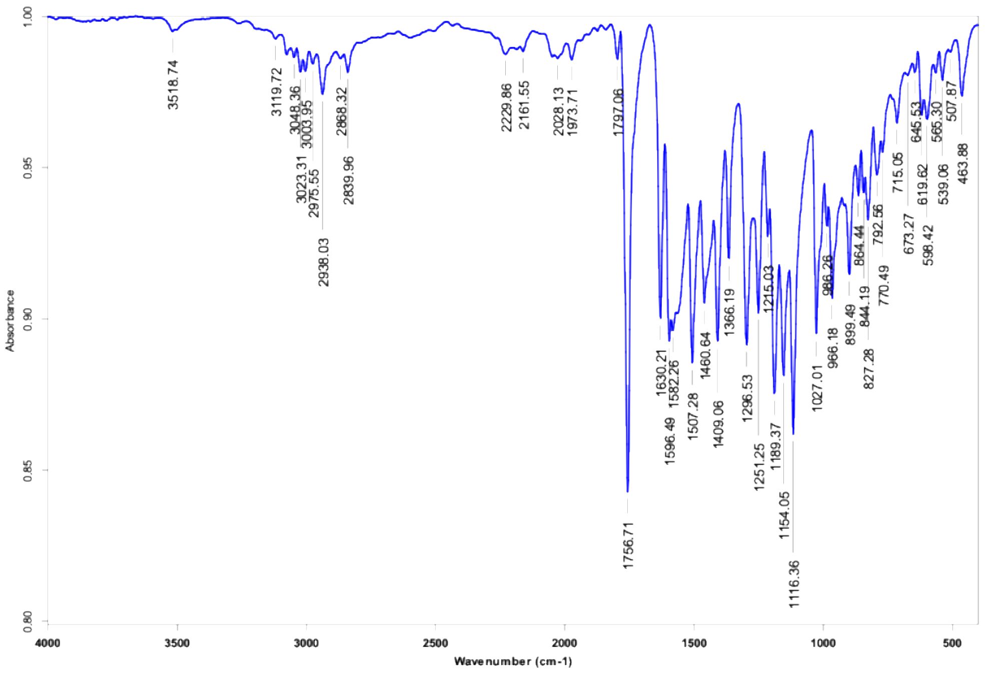

Figure S13. IR-ATR spectrum DAC (crystallization of ethyl acetate) at 2 °C. Corresponds to Form-1 and Form-3.

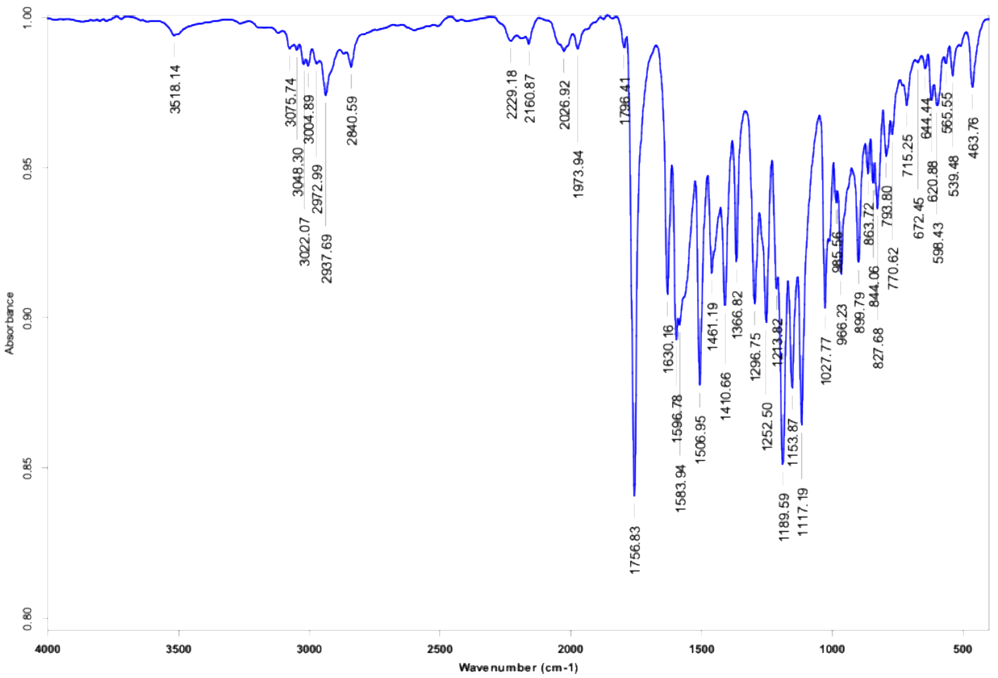

Figure S14. IR-ATR spectrum DAC (crystallization of dichloromethane) at room temperature. Corresponds to Form-1.

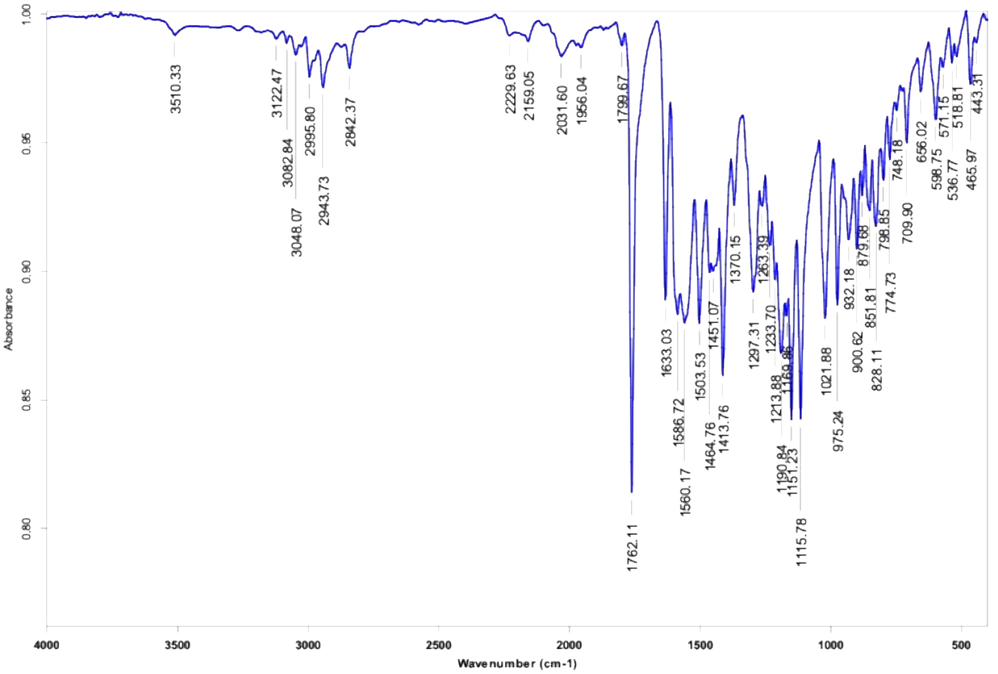

Figure S15. IR-ATR spectrum DAC (crystallization of dichloromethane) at 2°C. Corresponds to Form-3.

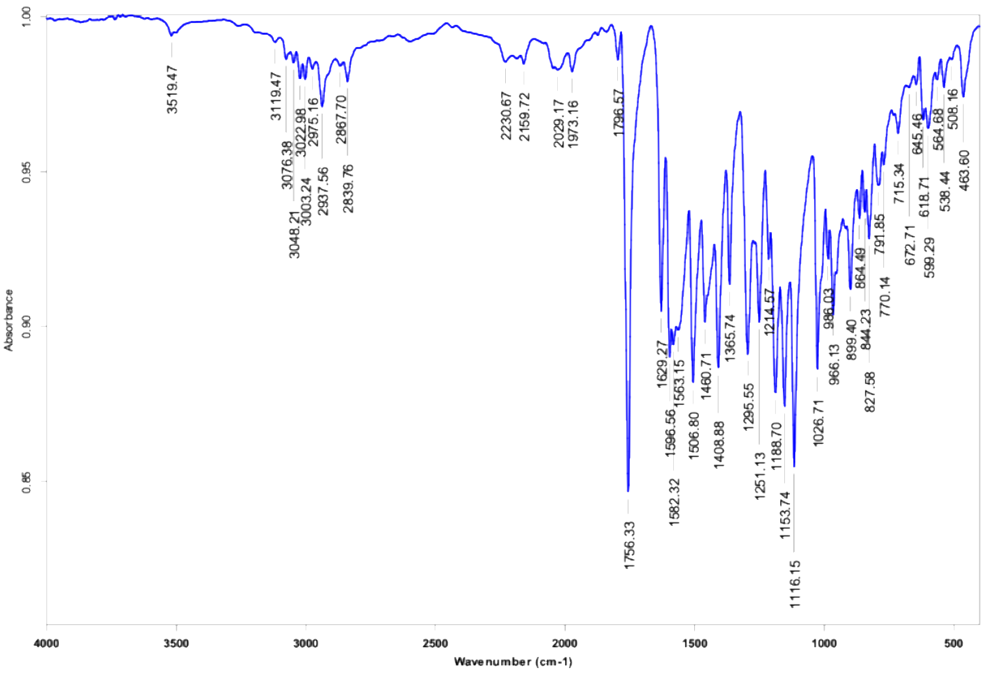

Figure S16. IR-ATR spectrum DAC (crystallization of acetone) at room temperature. Corresponds to Form-1.

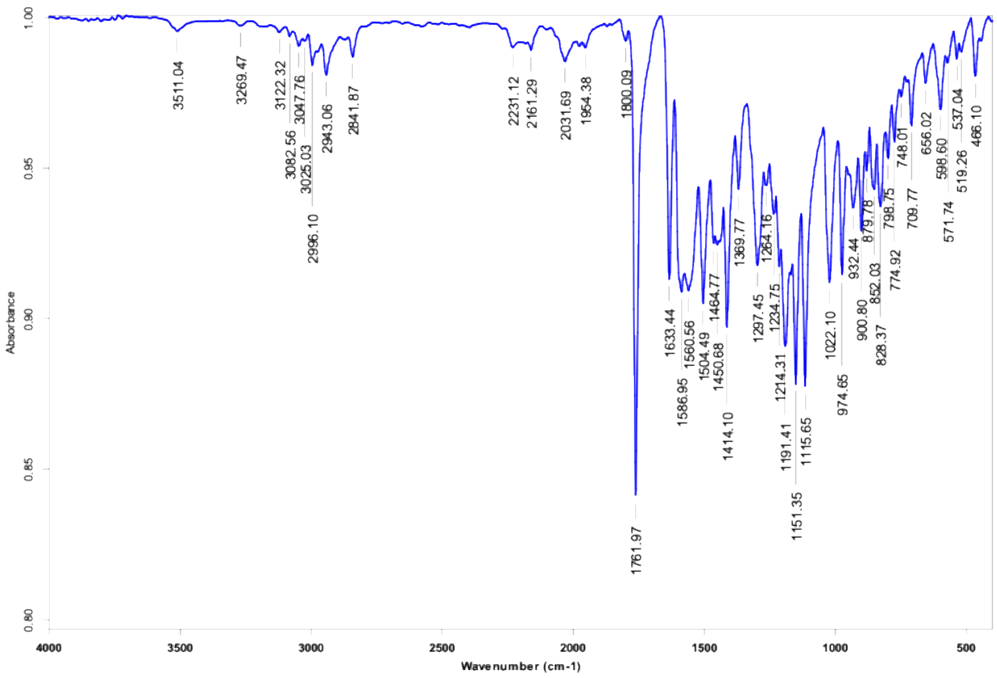

Figure S17. IR-ATR spectrum DAC (crystallization of acetone) at 2 °C. Corresponds to Form-3.

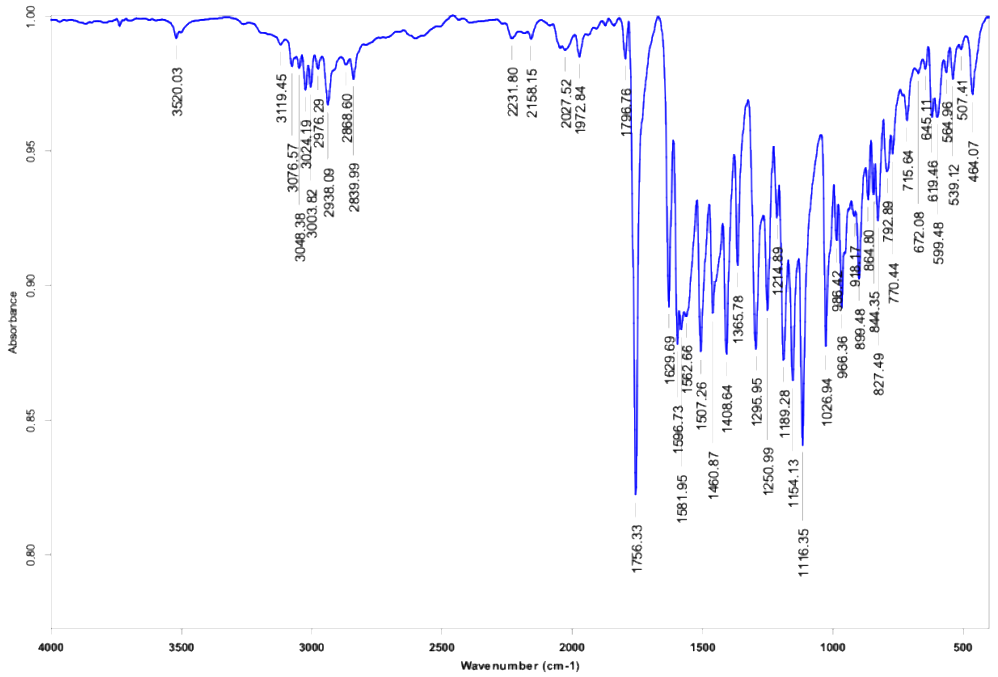

Figure S18. IR-ATR spectrum DAC (crystallization of ethanol) at room temperature. Corresponding to Form-1.

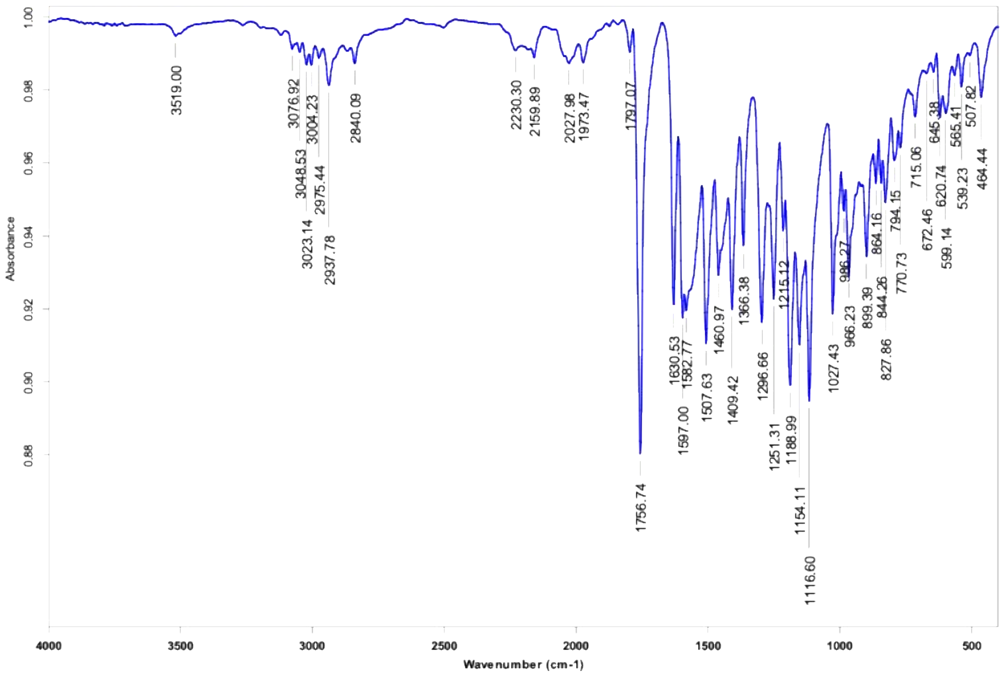

Figure S19. IR-ATR spectrum DAC (crystallization of ethanol) at 2 °C. Corresponds to Form-1.

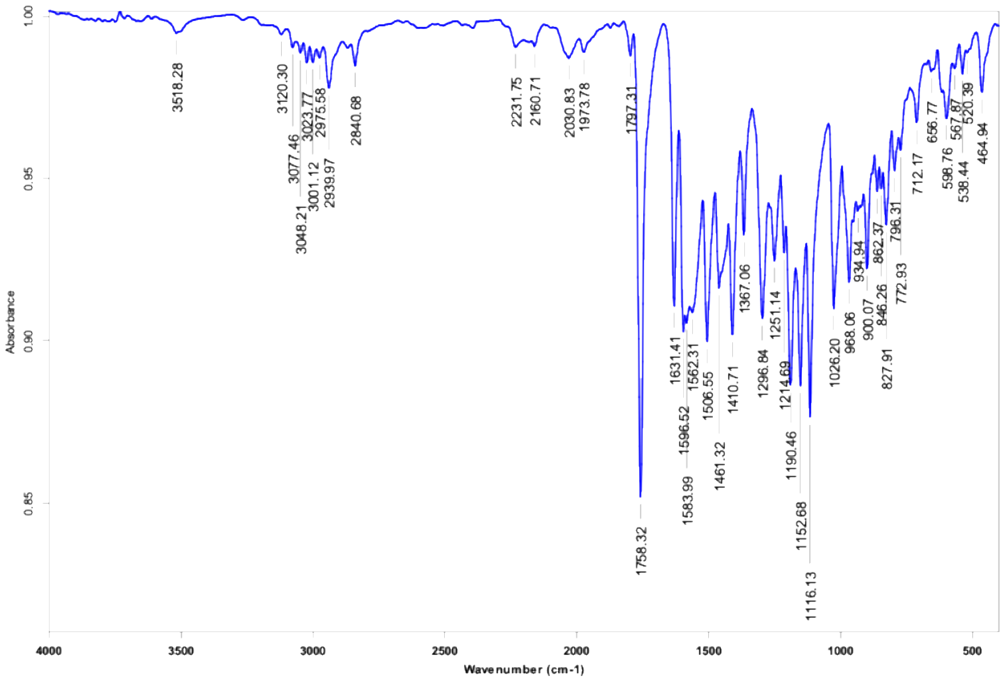

Figure S20. IR-ATR spectrum DAC (crystallization of methanol) at room temperature. Correspond to Form-1 and Form-3.

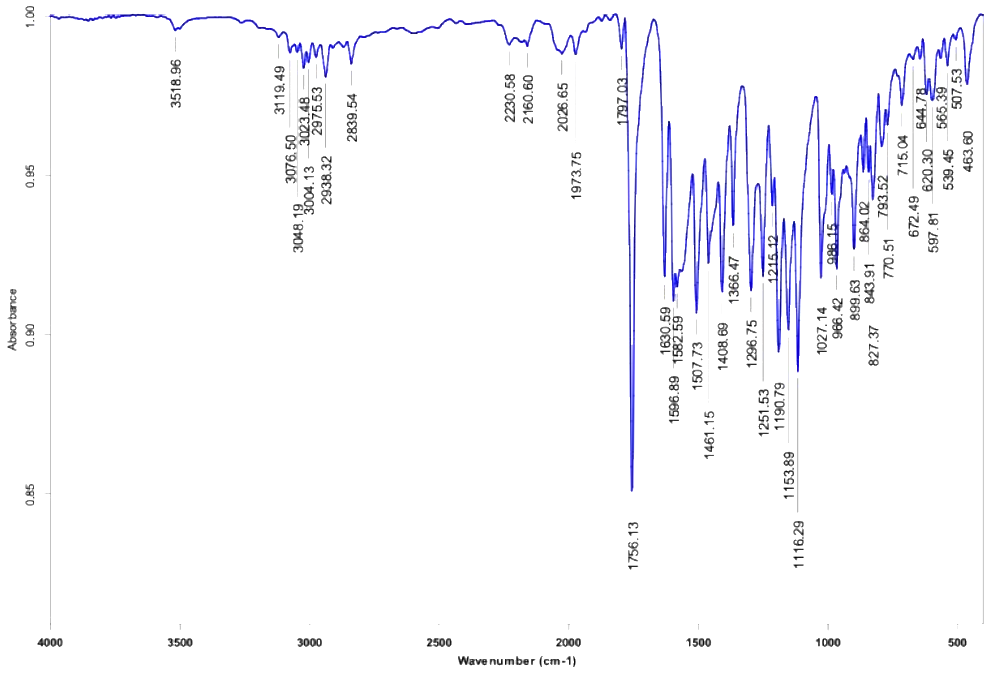

Figure S21. IR-ATR spectrum DAC (crystallization of methanol) at 2 °C. Corresponds to Form-1.

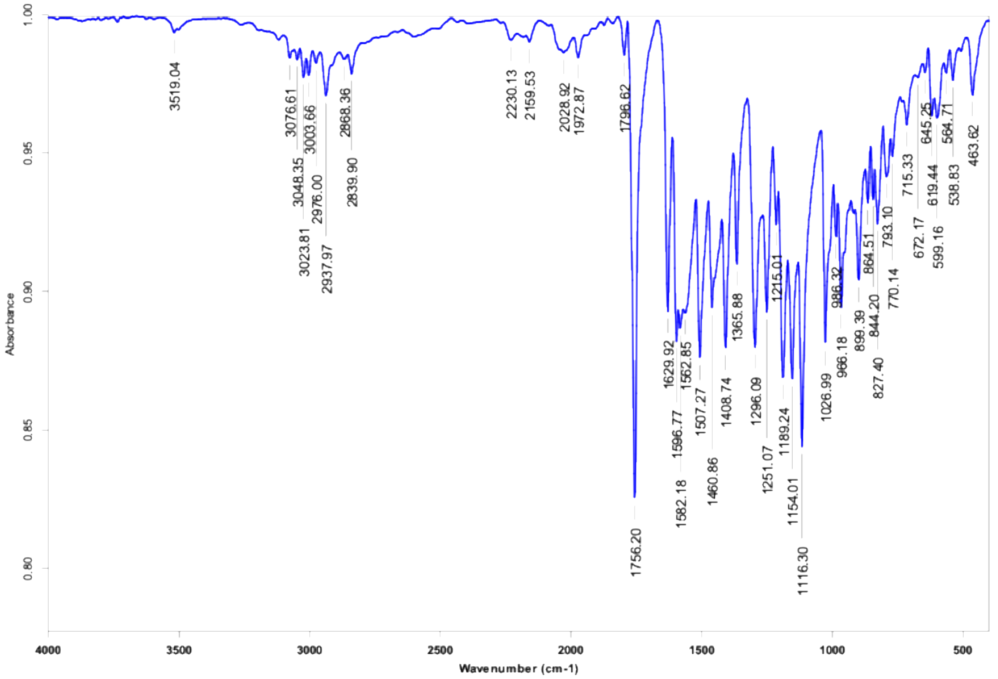

Figure S22. IR-ATR spectrum DAC (crystallization of acetonitrile) at room temperature. Corresponds to Form-1.

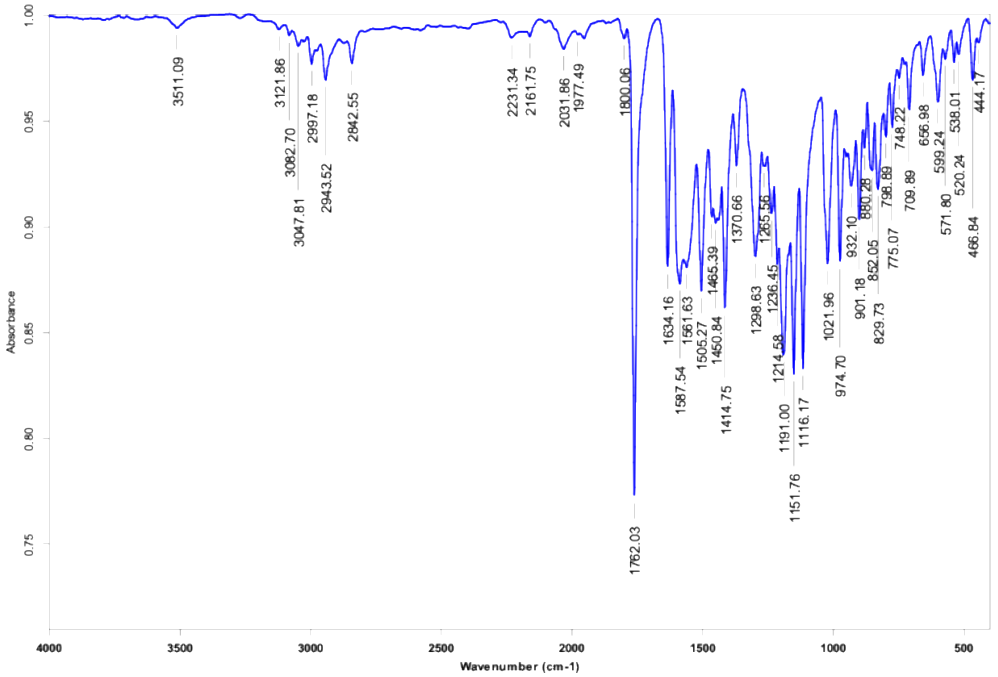

Figure S23. IR-ATR spectrum DAC (crystallization of acetonitrile) at 2 °C. Corresponds to Form-1 and Form-3.

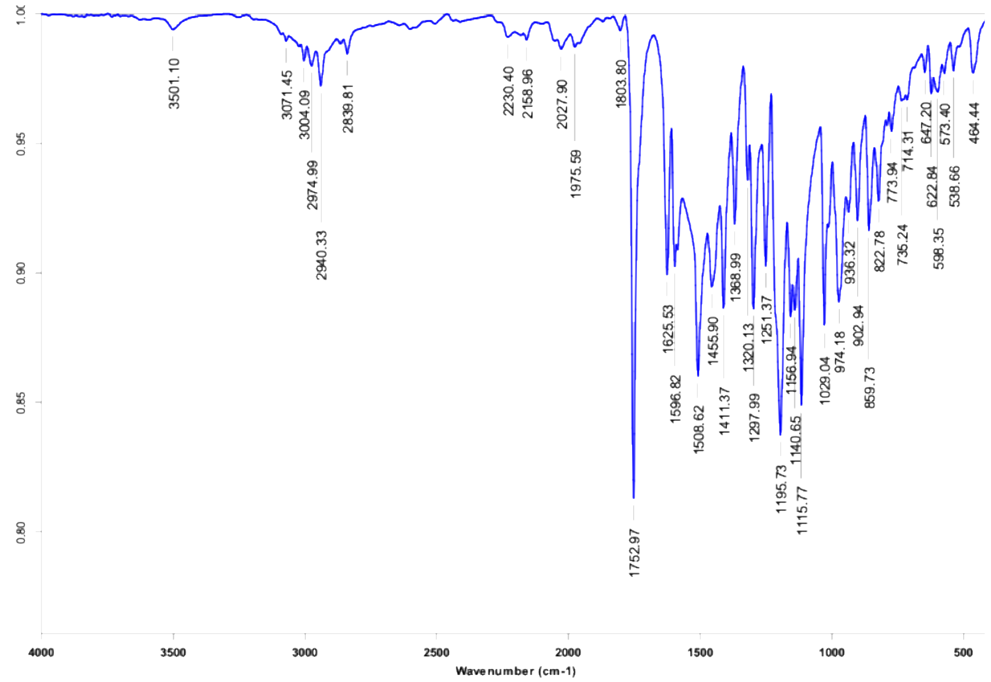

Figure S24. IR-ATR spectrum DAC (crystallization of ethyl acetate and hexane). Corresponds to Form-2.

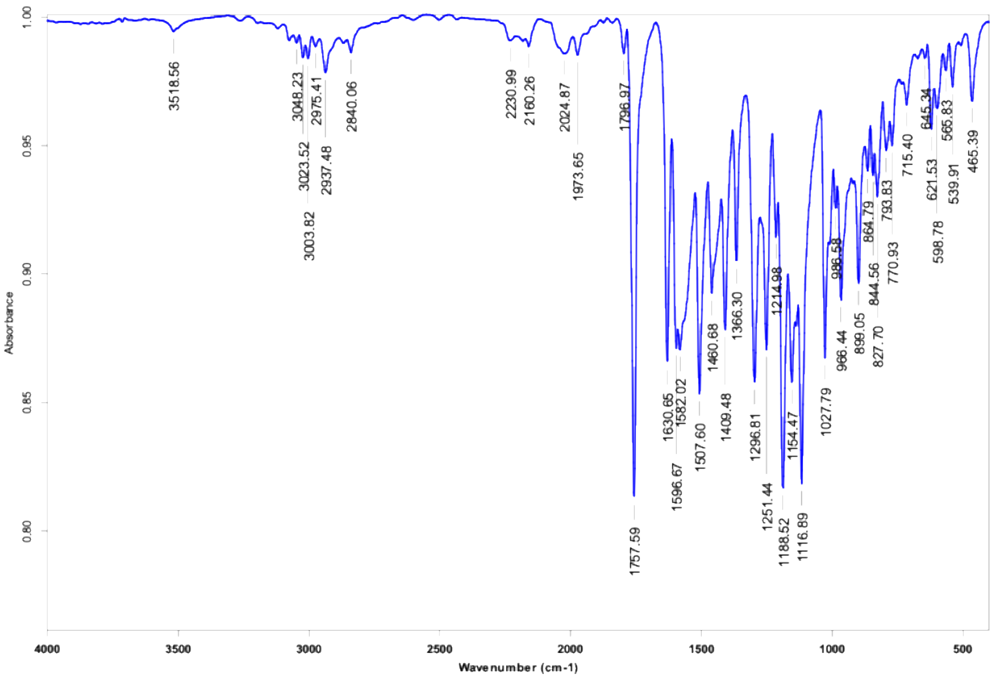

Figure S25. IR-ATR spectrum DAC (crystallization of dichlromethane and hexane). Corresponds to Form-1.

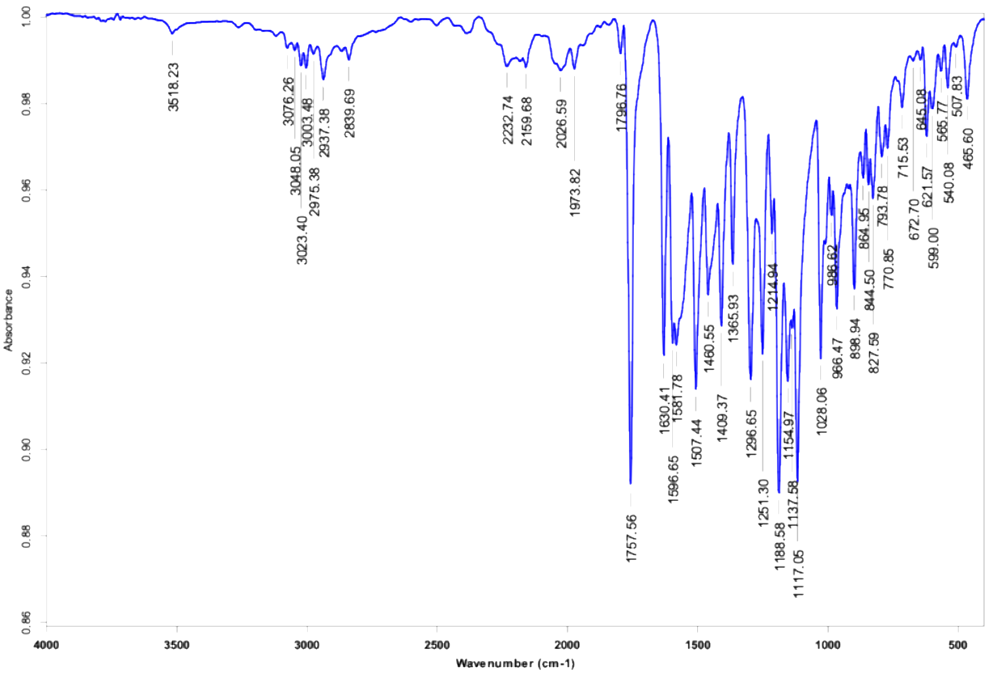

Figure S26. IR-ATR spectrum DAC (crystallization of acetone and hexane). Corresponds to Form-1.

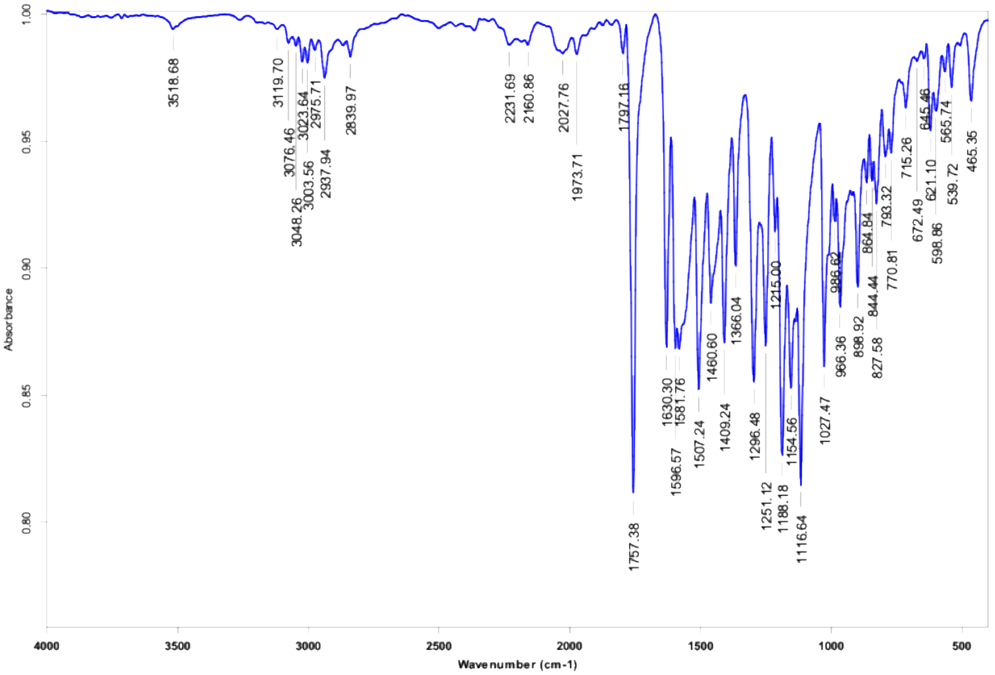

Figure S27. IR-ATR spectrum DAC (crystallization of ethanol and water). Corresponds to Form-1.

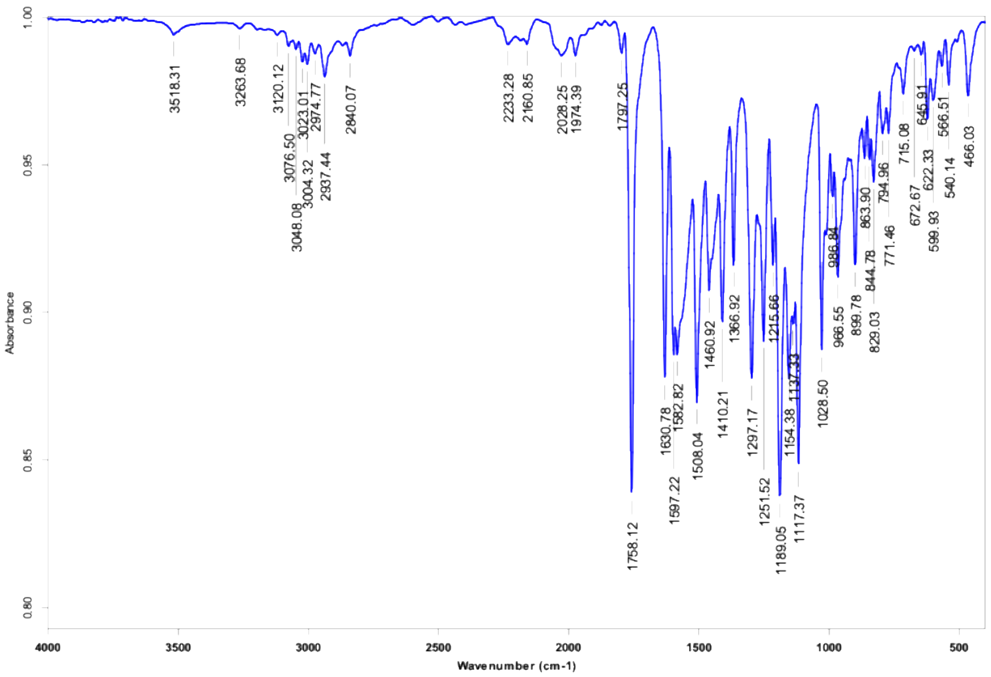

Figure S28. IR-ATR spectrum DAC (crystallization of methanol and water). Corresponds to Form-1.

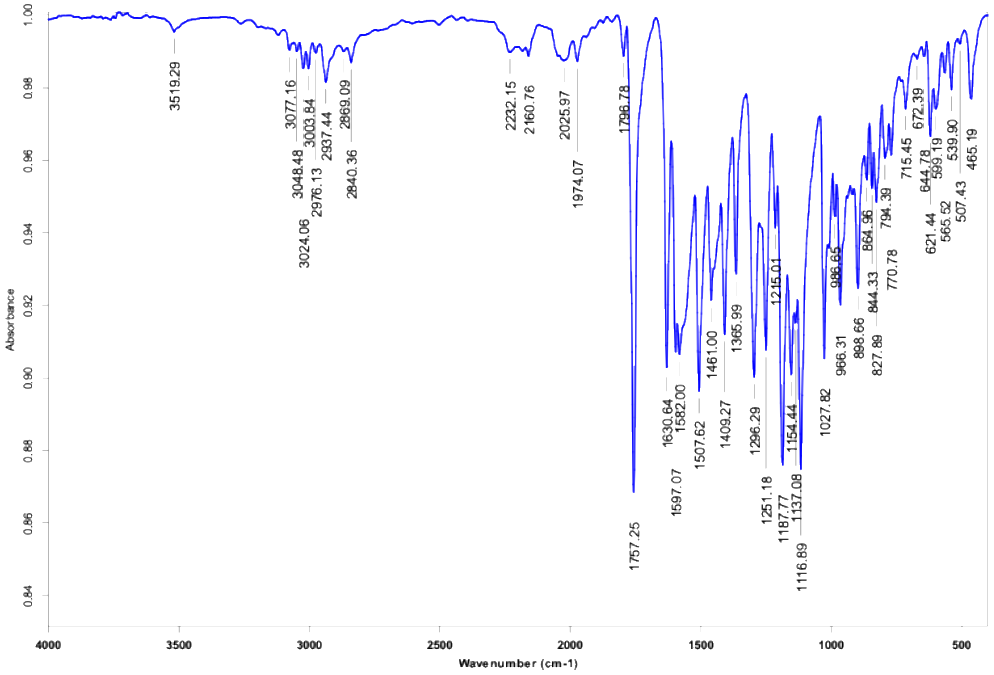

Figure S29. IR-ATR spectrum DAC (crystallization of acetonitrile and water). Corresponds to Form-1.

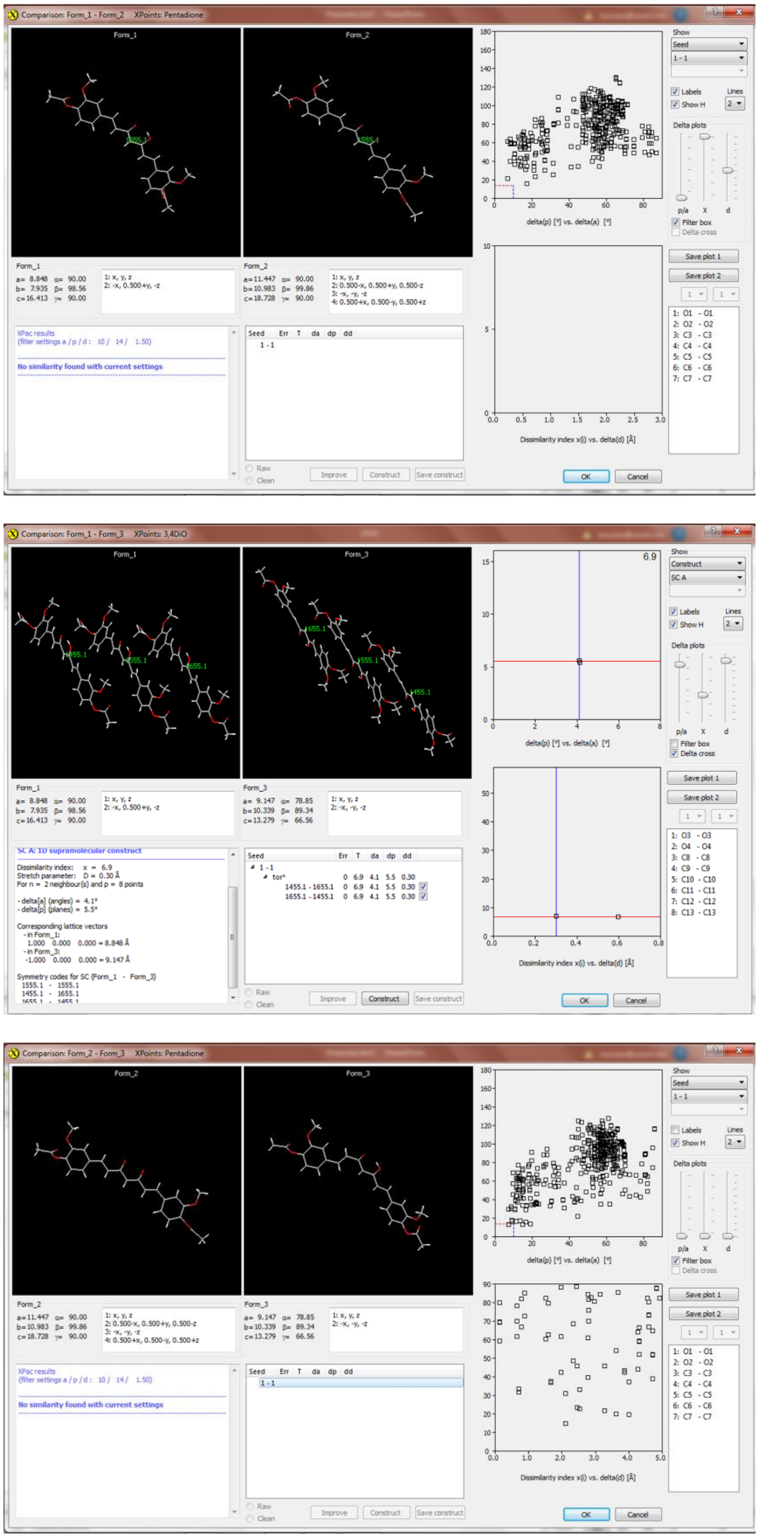

Figure S30. XPac windows for the comparison of the structure pairs Form 1–Form 2, Form 1–Form 3 and Form 2–Form 3.

Table S1. Crystal data and structure refinement of DAC polymorphs

**Identification code**	**Form-1**	**Form-2**	**Form-3**
Empirical formula	$C_{25}H_{24}O_8$	$C_{25}H_{24}O_8$	$C_{25}H_{24}O_8$
Formula weight	452.44	452.44	452.44
Temperature, K	298(2)	298(2)	298(2)
Wavelength	1.54178	0.71073	0.71073
Crystal system	Monoclinic	Monoclinic	Triclinic
Space group	$P2_1$	$P2_1/n$	P-1
a, Å	8.8478(2)	11.4469(19)	9.1473(2)
b, Å	7.9351(2)	10.9829(18)	10.3385(3)
c, Å	16.4135(4)	18.728(3)	13.2791(3)
α°	90	90	78.850(1)
β,°	98.5640(10)	99.863(3)	89.336(1)
γ,°	90	90	66.556(1)
Volume	1139.51(5) $Å^3$	2319.6(7)	1127.40(5)
Z	2	4	2
Density (calculated), Mg/m^3	1.319	1.296	1.333
Absorption coefficient, mm-1	0.824	0.097	0.100
F(000)	476	952	476
Crystal size, mm	0.458 x 0.131 x 0.057	0.384 x 0.164 x 0.088	0.422 x 0.422 x 0.182
Theta range for data collection, °	2.722 to 79.323	1.948 to 25.387	1.567 to 27.853
Index ranges	$-11 \le h \le 11$, $-9 \le k \le 10$, $-20 \le l \le 20$	$-13 \le h \le 13$, $-13 \le k \le 13$, $-22 \le l \le 22$	$-12 \le h \le 12$, $-13 \le k \le 13$, $-17 \le l \le 17$
Reflections collected	33815	23495	22613
Independent reflections	4828 [R(int) = 0.0465]	4255 [R(int) = 0.0495]	5336 [R(int) = 0.0321]
Completeness %	99.9	99.9	99.9
Refinement method	Full-matrix least-squares on F^2	Full-matrix least-squares on F^2	Full-matrix least-squares on F^2

Absorption correction	Semi-empirical from equivalents	Semi-empirical from equivalents	Semi-empirical from equivalents
Max. and min. transmission	0.6556 and 0.7506	0.8620 and 0.7980	0.6656 and 0.7456
Data / restraints / parameters	4828 / 1 / 305	4255 / 0 / 305	5336 / 0 / 305
Goodness-of-fit on F^2	1.079	1.018	1.027
Final *R* indices [*I*>2sigma(*I*)]	*R*1 = 0.0402, *wR*2 = 0.0948	*R*1 = 0.0542, *wR*2 = 0.1312	*R*1 = 0.0564, *wR*2 = 0.1466
R indices (all data)	*R*1 = 0.0481, *wR*2 = 0.1024	*R*1 = 0.0992, *wR*2 = 0.1538	*R*1 = 0.0689, *wR*2 = 0.1580
Absolute structure parameter	-0.04(8)	na	na
Largest diff. peak and hole, e.Å^{-3}	0.184 and -0.182	0.220 and -0.131	0.379 and -0.320

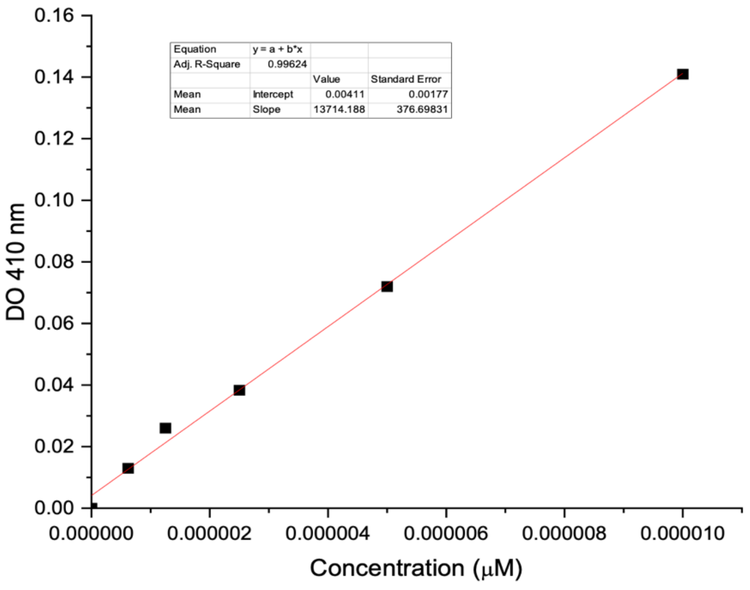

Figure S31. Curve standard of DAC in ethanol.

Polymorphous	Form-1	Form-2	Form-3
Crystal size, mm	0.458 x 0.131 x 0.057	0.384 x 0.164 x 0.088	0.422 x 0.422 x 0.182

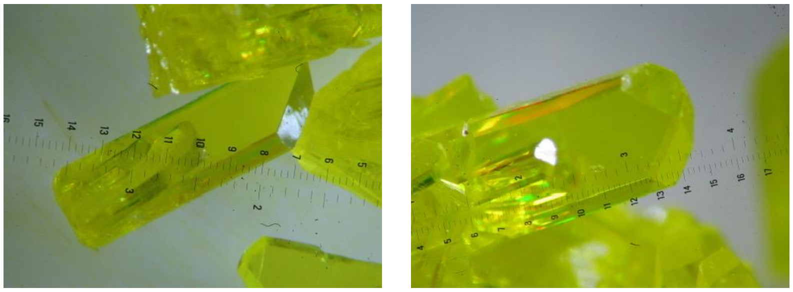

Form-1

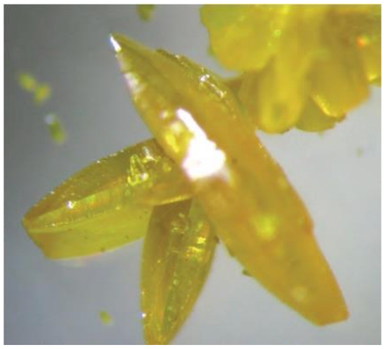

Form-2

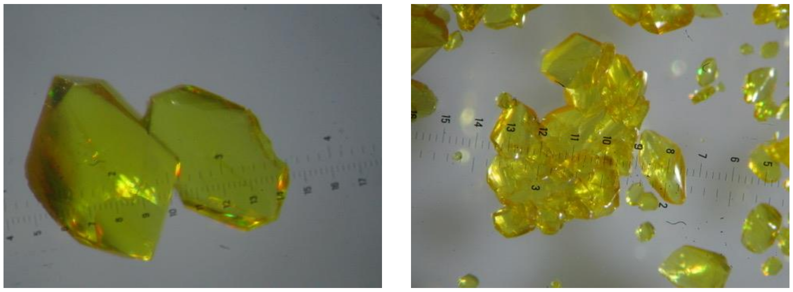

Form-3

Figure S32. Views of DAC polymorphs (under microscope) and size crystals obtained by X-Ray.

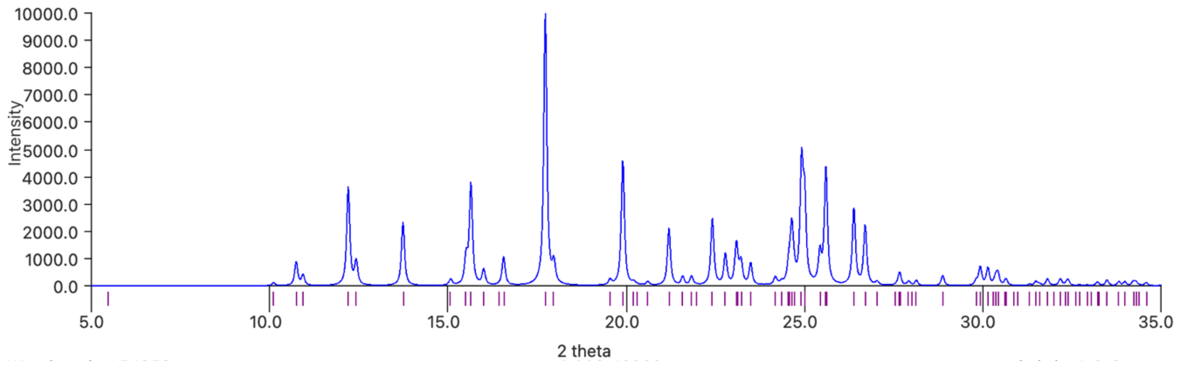

Form-1

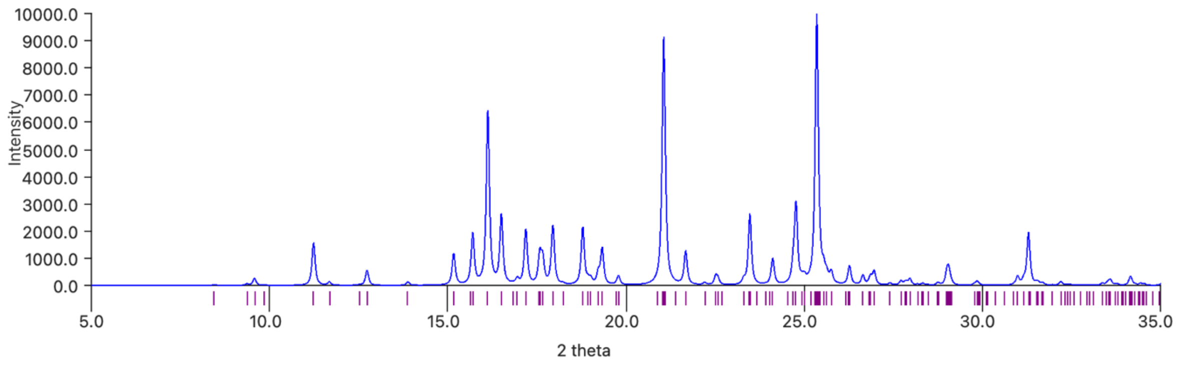

Form-2

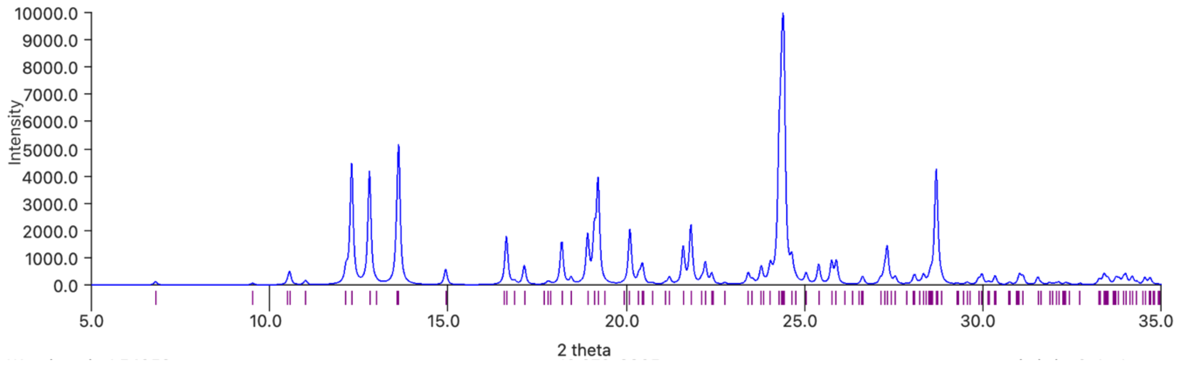

Form-3

Figure S33. Simulated PXRD powder X-ray diffraction patterns for the three polymorphs.
